# Valorization of the Isocyanate-Derived Fraction from Polyurethane Glycolysis by Synthesizing Polyureas and Polyamides

**DOI:** 10.1021/acssuschemeng.4c05482

**Published:** 2024-11-20

**Authors:** Jesus del Amo, Paula Bravo, Mennatallah M. Alashry, Juan Tejeda, Juan F. Rodríguez, Ana M. Borreguero

**Affiliations:** † Chemical Engineering Department, University of Castilla-La Mancha, Institute of Chemical and Environmental Technology, ITQUIMA, Avda. Camilo José Cela s/n, 13004 Ciudad Real, Spain; ‡ Chemistry Department, Faculty of Science, Mansoura University, 35516 Mansoura, Egypt; § Área de Química Orgánica, Facultad de Ciencias y Tecnologías Químicas, Universidad de Castilla-La Mancha, 13071 Ciudad Real, Spain

**Keywords:** glycolysis, flexible polyurethane foams, toluenediamine, polyureas, polyamides, circular economy

## Abstract

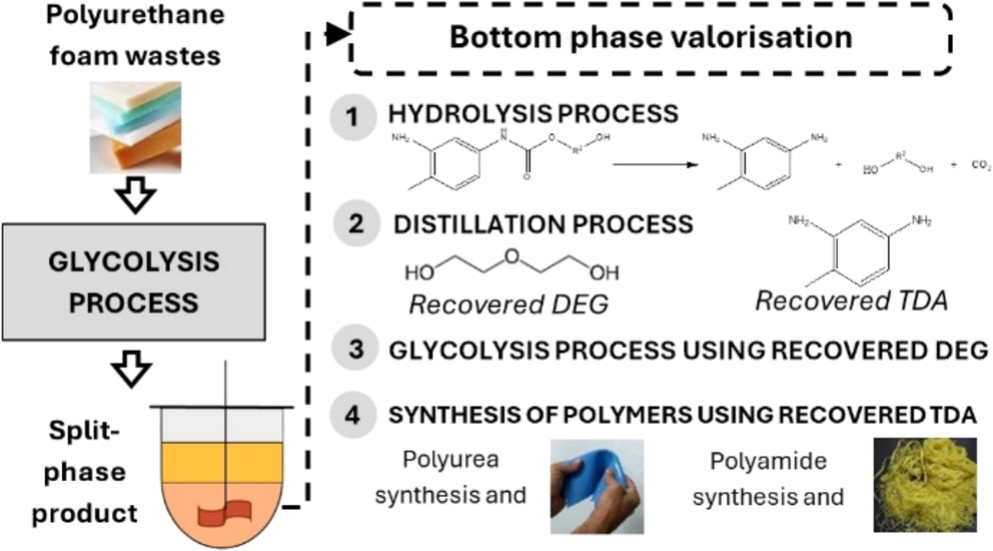

The isocyanate-derived fraction resulting as the bottom phase from the split-phase glycolysis of conventional polyurethane flexible foams has been given a new life based on the formation of amine-based polymers (polyureas and polyamides). For that purpose, the bottom phase was first hydrolyzed, producing toluenediamine and diethylene glycol, and further subjected to controlled vacuum distillation in order to recover both products separately. The hydrolysis reaction and the separation process conditions were determined and optimized, obtaining products with a purity comparable to that of commercial ones. Then, the recovered diethylene glycol was used in a new glycolysis process, obtaining a split-phase product with properties similar to those obtained using commercial diethylene glycol. Finally, the recovered toluenediamine was used in the synthesis of polyureas and polyamides. Both syntheses were modified with respect to the state of the art, replacing benzene with limonene in the synthesis of polyamides, which implies environmental improvements.

## Abstract 2

The research focuses on the sustainability of polyurethane foam recycling by valorizing all recovered products and synthesizing high-added-value products.

## 1 Introduction

Since the German scientist Otto Bayer discovered the polyaddition reaction between an isocyanate and a polyol to produce polyurethane (PU) in the early 1940s, the applications of PU have grown exponentially.^1−4^ The characteristic synthesis reaction of PUs is shown in Reaction 1.Polyurethane synthesis reaction.
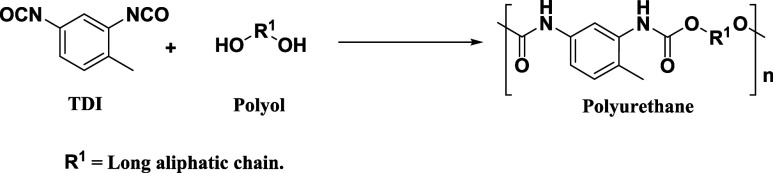
Reaction 1

For their synthesis, it is possible to find a wide range of monomers available and different processes, giving rise to materials with very different structures and properties.^5,6^ For this reason, due to their versatility, PU is one of the most widely produced polymers in the world, occupying the sixth–seventh place in the world production ranking, with a production of 27 million tonnes per year.^7−9^

PUs are primarily categorized as thermosets or thermoplastics. Thermoset PUs, the most common type, include foams, which can be further subdivided into flexible foams, which are used in car seats, mattresses, and packaging, and rigid foams, which are commonly found in building insulation, domestic refrigeration, and commercial refrigeration. On the other hand, thermoplastic PUs, often referred to as CASEs (Coatings, Adhesives, Sealants, and Elastomers), find application in construction, transportation, and marine applications due to their exceptional properties such as high wear and abrasion resistance, substantial tensile and tear strength, and significant damping capacity.^1,10^

As a result of their remarkable commercial success and widespread use, the amount of waste generated has increased significantly. This increase includes not only waste from products at the end of their life cycle but also rejects and cuts from routine production processes or products that have felt outside market specifications. In the past, PU waste was routinely landfilled. However, the significant volume of waste generated, coupled with the scarcity of available landfill space and the increase in environmental concerns, has led to a change in PU waste management practices. This change has moved toward recycling, which includes both physical and chemical methods.^11,12^ Physical recycling is the simplest recycling method, where PU waste is granulated and used as a filler or rebound for further applications in pillows or carpets. While these approaches provide a second life, the resulting application is typically of a lower added value than the original PU material. Therefore, chemical recycling is a more attractive option, as it allows the recovery of raw materials for the synthesis of new high-added-value products. The main chemical recycling methods include hydrolysis, phosphorolysis, aminolysis, and glycolysis, with the last one being the most advanced from a scientific and technological point of view, as well as more economically and industrially feasible.^4,11,13^

Glycolysis consists of a transesterification reaction between polyurethane and glycol to form polyol and reaction byproducts such as carbamates, primary amines, and carbon dioxide.^14−16^ The glycolysis reaction of polyurethane foams employing diethylene glycol is presented in Reaction 2.Polyurethane glycolysis reaction.
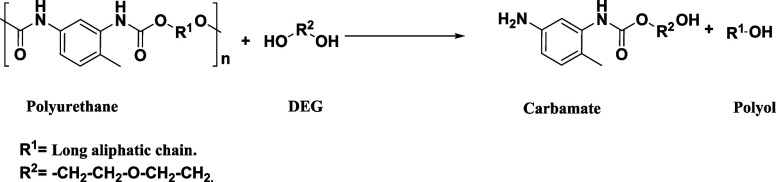
Reaction 2

Besides, the use of a large excess of glycol in the glycolysis reaction allows one to obtain, with most of the flexible foam residues, a split-phase product, where the upper phase is composed mainly of the recovered polyol and the bottom one contains mainly the isocyanate-related reaction byproducts and the excess of glycol. Therefore, the recovered product presents better properties than those obtained in single-phase processes. The applied glycolysis conditions were optimized in previous works of our group.^17^

This research will focus on expanding the reusing alternatives of the bottom-phase isocyanate-derived byproducts in high-added-value applications, closing the loop of the circular economy model for the PU life cycle. To the best of our knowledge, there are no previous works in the literature describing the recovery and valorization of the bottom-phase isocyanate-related compound, except for its use in the synthesis of rigid polyurethane foams, directly as a partial replacement of raw polyol or as an initiator in the synthesis of new polyols, and further use for rigid PU production.^18^

The novelty of this research focuses on the recovery of higher value products such as toluenediamine (TDA), which has been tested for its use in the synthesis of polyureas and polyamides, and diethylene glycol (DEG), which can be reused in the glycolysis process. The work involved the bottom-phase hydrolysis condition optimization and determination of the conditions for separating the two compounds (TDA and DEG) by distillation, obtaining both recovered products with high purity. In addition, several environmental improvements have been achieved in the synthesis of these materials, such as the replacement of benzene with limonene in the synthesis of polyamides, resulting in products with similar or even enhanced properties. On the other hand, the feasibility of using aromatic amines in the synthesis of aromatic–aliphatic polyamides with properties as those of aromatic polyamides or polyamides, which to the best of our knowledge have not been synthesized before, has been demonstrated. Therefore, products with a higher added value were obtained from the bottom phase of the glycolysis product compared to those obtained in previous research.

Finally, the recovered diethylene glycol was reused in a further glycolysis process, obtaining a product with properties identical to those obtained when using fresh glycol. Thus, one of the main drawbacks of the split-phase glycolysis process for polyurethane waste, consisting of the use of a large excess of glycol to induce phase separation and obtain products of higher purity, has been overcome, bringing the possible implementation of this process on an industrial scale closer in the near future.

## 2 Experimental Section

### 2.1 Glycolysis Reaction Process

Glycolysis reaction processes of flexible polyurethane foams were carried out at the laboratory scale, employing a 2 L volume reactor, which was heated by silicone oil from a thermostatic bath. Moreover, the reactor had at the top a condenser, a nitrogen intake to ensure an inert atmosphere and to avoid oxidation, an additional mouth to add glycol and the catalyst, and a stirring head to drive a six-blade Rushton-type agitator. It was also provided with a bottom valve, which was used to take samples during the reaction and for the discharge of the reaction product. The installation was placed in a fume extraction hood. Once a reaction temperature of 200 °C was reached, the polyurethane foam wastes were fed by an automatic feeder for 1 h. The stirring speed was 300 rpm to ensure complete homogenization. The reaction conditions employed were optimized previously, which are a ratio of PU to glycolysis agent of 1:1, a catalyst concentration of 0.1 wt %, a feeding time of 1 h, and a reaction time of 3 h.^17^ After the reaction time, the glycolysis product was extracted and left to decant in a funnel to separate the different phases.

### 2.2 Hydrolysis Process

The hydrolysis reaction of the bottom phase of the glycolysis product was carried out on a 5 L stainless steel pressure reactor, thermostated with silicone oil from a circulation thermostat, with an operating temperature range of −60 to 200 °C. The reactor had a pressure and temperature indicator to control the reaction conditions. Besides, the reactor presented a stirring head to drive a four-blade agitator, which worked at a high speed to ensure complete homogenization. For the discharge of the product, there was a lower tap at the bottom of the reactor vessel. The experimental hydrolysis procedure consisted of feeding the reactor with a mixture of basic water (pH higher than 12) and the bottom phase obtained from the glycolysis process, with a mass ratio of 1:1. The temperature reaction was 200 °C, and the reaction time was 3 h. The hydrolysis product was placed in an oven at 100 °C for 24 h to remove the water.

### 2.3 Distillation and Purification Processes

A first separation study of the hydrolysis product compounds was carried out in an Aldrich Kugelrohr short-pass distiller, which is specially designed to separate high-boiling-point compounds, allowing operation up to temperatures of 220 °C. From the digital control panel of the equipment, it was possible to adjust the temperature and the rotation speed of the flask. In addition, this installation was equipped with a vacuum pump, a vacuum trap, and a vacuum controller. Different temperature and pressure conditions were tried to determine the separation steps.

**Figure 1:**
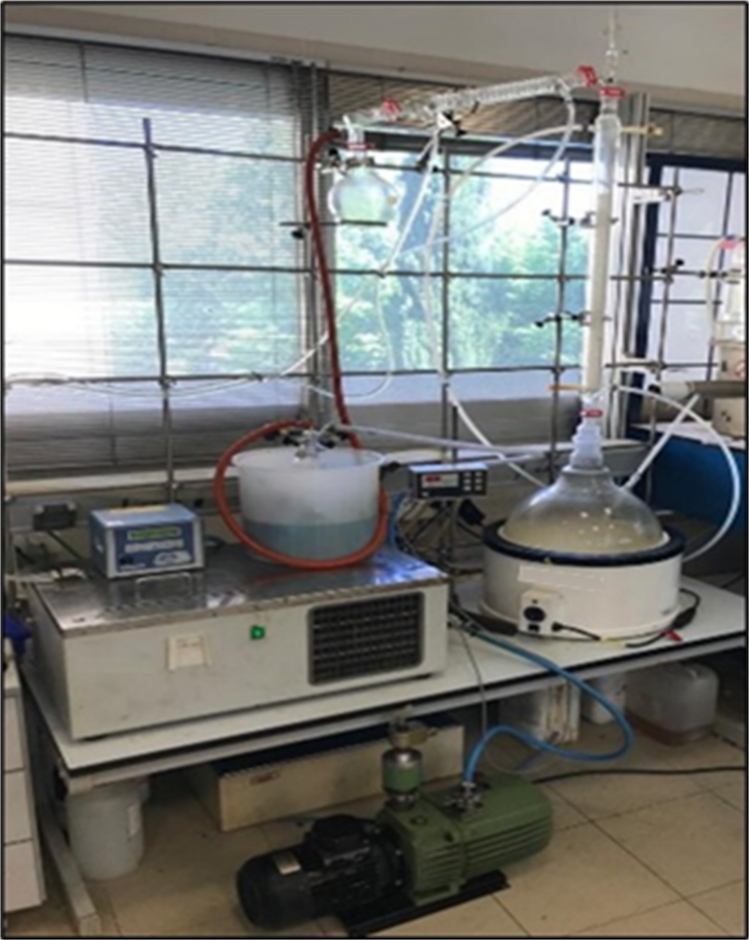
Distillation unit.

Once the separation conditions of the hydrolysis product were determined, the process was carried out on a larger scale using a 20 L flask, which was thermostated with a heating mantle with a sensor for temperature control and connected to a glass column thermostated with silicone oil. The condenser located at the head of the column was cooled by means of a thermostatic bath with monoethylene glycol and connected to a 1 L flask where the distillate was accumulated. Finally, the installation had a thermometer at the head of the column and a high vacuum pump, a vacuum trap, and a vacuum controller to achieve the ideal operating conditions for the separation of the different products. Figure 1 shows the distillation unit.

### 2.4 Polyurea Synthesis Process

Polyurea samples are synthesized according to the methodology reported in previous work.^19^ The synthesis process started by dissolving toluenediamine (TDA, 5 mmol, 0.61 g) and 0.175 g of LiCl in 15 g of dimethylacetamide (DMAc). Then, isocyanate was added to the reaction mixture and stirred for 15 min. Then, the reaction mixture was stirred under a nitrogen atmosphere for 4 h to provide a viscous liquid, and after that, the solution was cast in molds. The molds were kept in an oven at 50 °C for 48 h. 5 mmol of three different isocyanates were used, namely, HMDI (0.84 g), isophorone diisocyanate (1.11 g), and TDI (0.87 g).

### 2.5 Polyamide Synthesis Process

Generally, polyamides are synthesized by a condensation reaction between amines and carboxylic acids or their derivatives. In this work, polyamide synthesis was carried out using two different methodologies, namely, single-phase synthesis and two-phase synthesis.

In the case of single-phase synthesis, a previously reported methodology was followed.^20^ The process consisted of dissolving (0.0125 mol, 1.525 g) toluenediamine (TDA) in 50 g of dimethylacetamide (DMAc) for 30 min and then adding 0.0125 mol of acid chloride (2.54 g of isophthaloyl chloride (aromatic polyamide polymer) or 2.29 g of adipoyl chloride (aromatic–aliphatic polyamide polymer)), depending on the desired polymer. The reaction was kept under a nitrogen atmosphere for 24 h, and then ethanol was poured in, precipitating the polymer. Finally, the solid was filtered, washed several times with ethanol to remove any remaining starting material, and dried in an oven at 50 °C for 24 h.

The two-phase procedure for the synthesis of polyamides is based on previous research.^21^ The synthesis starts by preparing a solution of TDA (0.02 mol, 2.44 g) in a mixture of 48.4 mL of acetone, 151.6 mL of water, and 1.60 g of sodium hydroxide. On the other hand, 0.03 mol of acid chloride (6.09 g of isophthaloyl chloride (aromatic polyamide) or 5.49 g of adipoyl chloride (aromatic–aliphatic polyamide)) is dissolved in a solution of 16.6 mL of acetone and 33.4 mL of benzene or limonene, highlighting the advantage of replacing benzene with limonene as it is a green solvent, which implies an environmental improvement. The second solution is then poured into the aqueous solution and stirred for 5 min, forming a precipitate. This precipitate is filtered, washed with ethanol, and dried in an oven at 50 °C for 24 h.

## 3 Results and Discussion

### 3.1 Hydrolysis Reaction of the Bottom Phase of the Glycolysis Product

After the glycolysis process employing conventional polyurethane foam wastes and the reaction conditions indicated in Section 2.2, a biphasic product was obtained. The upper phase was composed mainly of the recovered polyol (with approximately 80 wt % purity), and the bottom phase was composed of the reaction byproducts and the excess of glycol (with a composition of approximately 65 and 35 wt %, respectively) together with slight losses of recovered polyol solubilized in this phase (about 1 wt %).

The bottom phase was hydrolyzed to transform the reaction byproducts, mainly carbamates, into primary amines, such as toluenediamine (TDA) and diethylene glycol (DEG). The reaction of the hydrolysis process is presented in Reaction 3.Hydrolysis process reaction.
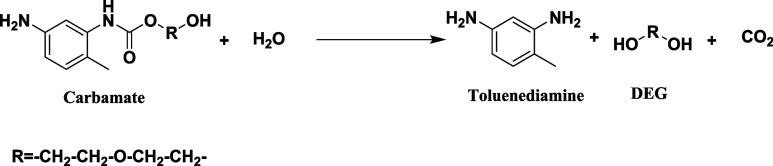
Reaction 3

The hydrolysis reaction conditions were as follows: a mass ratio of the bottom phase to basic water (pH higher than 12) of 1:1, a pressure close to 16 bar, a reaction temperature of 200 °C, and a reaction time of 3 h. The product obtained after the hydrolysis reaction was placed in an oven at 100 °C for 24 h to remove the water.

Infrared analyses were carried out to characterize the bottom phase, and the product was obtained after the hydrolysis reaction and dried in an oven. Figure S1 presents these results.

The spectra of the bottom phase and hydrolysis product presented the same signals, but the signal corresponding to carbamates C=O at 1712 cm^–1^ was observed in the spectra of the bottom phase, which did not appear in the spectra of the hydrolysis product.^22^ In addition, the signal of the amine groups –NH at 1624 cm^–1^ presented a higher intensity in the spectra of the hydrolysis product.^22^

Finally, from these results, it can be concluded that the hydrolysis reaction was successful, converting the carbamates into diethylene glycol and toluenediamine, since the signal corresponding to the carbamates did not appear in the FTIR spectra.

### 3.2 Separation and Characterization of the Product Compounds after the Hydrolysis Reaction

Once the hydrolysis process was carried out, the product obtained was dried in an oven at 100 °C for 24 h to remove the water. Then, the objective was to separate the different compounds present in the dehydrated hydrolysis product, recovering DEG and toluenediamine. The separation tests were carried out on a smaller scale unit, where the operating conditions were optimized for further application on a larger scale.

**Figure 2:**
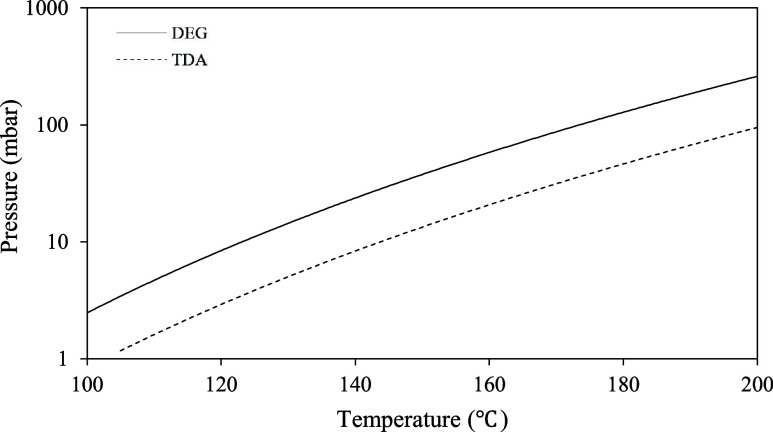
Vapor pressures of diethylene glycol and toluenediamine.

For the optimization of the separation conditions, it was necessary to theoretically determine the vapor pressures at different temperatures for the main compounds (TDA and DEG). Figure 2 shows the vapor pressures of diethylene glycol and toluenediamine, calculated using Aspen HYSYS commercial software.

Using the short-pass distiller described in Section 2.4, the hydrolysis product was distilled in different stages. The temperature was fixed at 160 °C, and the pressure was varied. In the first stage, the pressure was set at 55 mbar to recover diethylene glycol; in the second stage, the pressure was set at 18 mbar to ensure the complete removal of diethylene glycol, although with impurities of toluenediamine; and in the third stage, 10 mbar was set to recover toluenediamine with high purity. In addition, the distillation residue contained higher-molecular-weight compounds that were not valorized. The recovered toluenediamine was a solid product and was further purified by recrystallization. This purification consisted of two washes, one with cold ethanol, followed by vacuum filtration to obtain the recovered toluenediamine with a purity close to 100 wt %.

Once the working conditions were established at a small scale, the distillation unit described in Section 2.4 was used to obtain a larger quantity of recovered products. It is worth pointing out that the distillation residue was only approximately 1 wt %. Figure S2 shows the GPC chromatograms of the recovered products and the distillation residue compared to those of pure diethylene glycol and TDA.

These results showed that in the first distillation stage, it is possible to recover diethylene glycol with almost 100 wt % purity, as evidenced by a single peak in GPC with the same retention time as pure DEG. During the second distillation at 18 mbar, two peaks were observed in GPC, indicating the removal of the remaining DEG and some amine impurities TDA. Analyses of the product from the third distillation step at 10 mbar showed a single peak with the same retention time as commercial toluenediamine, indicating the successful recovery of high-purity toluenediamine.

Infrared analyses of the products from the different distillation stages were carried out to confirm the GPC results (Figure S3).

The spectra of the distilled product at 55 mbar showed the same structure and the same intensity as the spectra of DEG. The main signals were those corresponding to OH groups at 3340 and 2900 cm^–1^ to CH groups, which are the main signals of diethylene glycol.^22^ On the other hand, the result of the distilled product at 18 mbar showed the signals corresponding to diethylene glycol but with the amine group peak that appears at 1624 cm^–1^. These conditions allowed the removal of the rest of the diethylene glycol but with the presence of toluenediamine, since the spectra from the product at 10 mbar do not present the DEG characteristic group peaks.^22^ The last two spectra correspond to the comparison between pure and recovered toluenediamine. Both spectra showed the same signals and intensities, confirming the high purity of recovered TDA.

Furthermore, the ^1^H NMR of the separated TDA ^1^H NMR (500 MHz, CDCl_3_-*d*) δ 6.83 (d, *J* = 8.0 Hz, 1H) corresponds to the aromatic proton in the ortho position to the methyl group of TDA, 6.09 (dd, *J* = 7.9, 2.3 Hz, 1H) corresponds to the aromatic proton in the ortho position to the amine group and the other aromatic proton of TDA, 6.06 (d, *J* = 2.3 Hz, 1H) corresponds to the aromatic proton located between the two amino groups, 3.46 (s, 4H) corresponds to the four protons of amino groups, and 2.07 (s, 3H) corresponds to the methyl group of TDA. The spectra are shown in Figures S4 and S5.

On the other hand, based on 1 kg of PU, 0.06 kg of DEG is consumed in the glycolysis reaction, and depending on the composition of PU, 0.24 kg of TDA can be obtained per kg of residue. Since in the distillation process, 0.89 and 0.2 kg of recovered DEG and recovered TDA, respectively, were obtained per kg of hydrolyzed bottom phase, recovery yield values of 95% for DEG and 84% for TDA were obtained.

As a general conclusion of this section, it should be highlighted that the feasibility of recovering value-added products such as DEG and toluenediamine from the bottom phase of PU foam glycolysis, which was previously considered as waste or with poor valorization, has been demonstrated. Consequently, the interest in glycolysis has been increased by adding the recovery of other materials to the possible recovery of polyol, transforming it into a global circular economy model with economic and environmental improvements.

### 3.3 Glycolysis Reaction Employing the Recovered Diethylene Glycol

The recovered diethylene glycol obtained after the distillation process was used in a new glycolysis process using the reaction conditions described in Section 2.2. After the reaction time, the product obtained showed a split phase, which was separated by decantation.

Figure S6 shows the GPC chromatograms of both phases together with the chromatograms of pure polyol and diethylene glycol.

From these results, the concentration of each compound in the different phases can be estimated.^23^ These results are presented in Table 1.

**Table 1:** Concentrations of the Different Compounds in the Upper and Bottom Phases

	concentration (wt %)	
component	upper phase	bottom phase
recovered polyol	83.9	1.8
reaction byproducts	12.9	74.4
diethylene glycol	3.2	23.8

These results showed the feasibility of the glycolysis process using the recovered diethylene glycol instead of the commercial one, since an upper phase with a recovered polyol purity of higher than 80 wt % has been obtained, which after cleaning could replace raw polyol in the synthesis of new polyurethane foams. Besides, the absence of oligomers in the glycolysis products indicates a complete breakdown of the polyurethane backbone into polyol and reaction byproducts. On the other hand, the bottom phase presented slight losses of recovered polyol solubilized; this phase is mainly composed of reaction byproducts and excess glycol. In conclusion, the recovery and further reuse of the recovered diethylene glycol have been demonstrated, improving the circularity of the process with economic and environmental feasibility.

### 3.4 Application of Recovered Toluenediamine in the Synthesis of New Materials

#### 3.4.1 Synthesis and Characterization of Polyureas

Polyureas were synthesized according to the method indicated in Section 2.5(19) Three different isocyanates, namely, hexamethylene diisocyanate (HMDI), isophorone diisocyanate (IPDI), and toluene diisocyanate (TDI), were used in the synthesis, together with the recovered toluenediamine. Reaction 4 represents the chemical reactions of the synthesized polyurea.Synthesis of different polyurea implementing different aliphatic and aromatic diisocyanates.
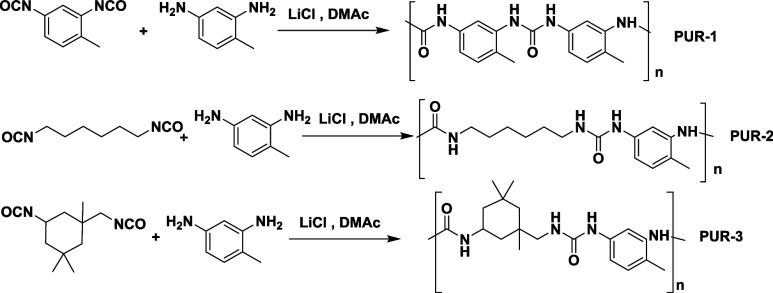
Reaction 4

The FTIR of the polyurea is done on a film basis. From the spectra in Figures S7 and S8, it is possible to appreciate that the polyurea is formed due to the characteristic signals for the disappearance peaks at 2246, 2240, and 2226 cm^–1^ corresponding to the isocyanate group (–NCO) of IPDI, HMDI, and TDI respectively. There is a shift of N–H from 3400 cm^–1^ in the amine to 3312, 3326, and 3332 cm^–1^ in PUR-1, PUR-2, and PUR-3, respectively. This is because of the involvement in the hydrogen bond formation.^24^ There are two characteristic peaks to the urea linkage, which are amide I (C=O) and amide II (CO–N–H).^25^ These two peaks appeared at 1626 and 1552 cm^–1^ in the case of PUR-1, 1632 and 1566 cm^–1^ for PUR-2, and 1630 and 1560 cm^–1^ for PUR-3. Furthermore, the stretching vibration of the carbonyl group at 1626–1632 cm^–1^ and the stretching vibration of the N–H group at 3312–3332 cm^–1^ indicate that the hydrogen bonds formed between the polyurea chains in the solid polymer are mostly ordered hydrogen bonds.^25,26^ In all polyurea cases, both aliphatic and aromatic C–H stretching appears from 2856 to 2952 cm^–1^ and 3042 to 3052 cm^–1^, respectively.

The structures of the synthesized polyurea were confirmed by ^1^H NMR. Due to the low solubility of the synthesized polyurea, the NMR analysis was done at 90 °C.

For PUR-1 (Figure S9), ^1^H NMR (500 MHz, DMSO-*d*_6_) δ 7.94–9.29 (m, 4H) corresponds to the protons of the urea linkage, 7.03–7.7.87 (m, 6H) corresponds to the aromatic protons from TDA and TDI incorporated in the structure, and 2.23 (s, 6H) corresponds to the two methyl groups of TDI and TDA incorporated in the structure.

For PUR-2 (Figure S10), ^1^H NMR (500 MHz, DMSO-*d*_6_) δ 8.15(1H) corresponds to the one proton of the urea linkage, 6.93–7.64 (m, 3H) corresponds to the aromatic protons of TDA incorporated in polyurea, 5.66–6.91 (m, 3H) corresponds to the three amino groups in the urea linkage, 5.66, 2.82 around (4H) near to the water of DMSO corresponds to the two terminal methylene groups of the HMDI part of polyurea, 2.11 (s, 3H) corresponds to the methyl group of TDA, and 1.36–1.46 (m, 8H) corresponds to the four internal methylene groups of the HMDI part in polyurea.

For PUR-3 (Figure S11), ^1^H NMR (500 MHz, DMSO-*d*_6_) δ 8.10 (1H) corresponds to the one proton of the urea linkage, 7.06–7.71 (m, 3H) corresponds to the aromatic protons of TDA incorporated in polyurea, 5.79–6.92 (m,3H) corresponds to the three amino groups in the urea linkage, 3.81 (s, 1H) corresponds to the proton of the CH group of the aliphatic part, 2.12 (s, 3H) corresponds to the methyl group of TDA, 1.20–1.80 (m, 4H) corresponds to two methylene groups of the aliphatic part, and 0.94–1.06 (m, 12H) corresponds to the three methyl groups in the aliphatic part.

Once the feasibility of synthesizing polyureas with the recovered TDA using the different isocyanates was demonstrated, the thermal characterization of the synthesized products was carried out. The results are presented in Table 2.

**Table 2:** Thermal Analysis of the Synthesized Polyurea Obtained from a) DSC and b) TGA

polymer	T_m_^a^ (°C)	T_10%_^b^ (°C)	T_20%_^b^ (°C)	T_50%_^b^ (°C)	char amount at 700^b^ °C (%)
PUR-1	170	208	241	346	9.31
PUR-2	182	177	220	335	12.85
PUR-3	218	199	233	355	11.93

The TGA showed multistage decomposition (Figure S12), which agrees with the results in the literature.^27,28^ All of the synthesized polyureas showed three weight loss/decomposition stages. The first one corresponds to the 10% weight loss until 200 °C.^29^ For PUR-1, the material is characterized by the onset of weight loss at 200 °C and the maximum decomposition rate at 311 °C. For PUR-2, the material is characterized by the onset of degradation at 240 °C and the maximum decomposition rate at 317 °C. For PUR-3, the material is characterized by the onset of degradation at 237 °C and the maximum decomposition rate at 348 °C. Finally, the third weight loss stage, corresponding with the degradation temperature of polyureas, is usually higher than 350 °C.^19,30,31^ Finally, the third weight loss stage, corresponding to the degradation temperature of polyureas, is usually higher than 350 °C.^19,30,31^ However, as shown in previous studies, the incorporation of aromatic amines resulted in materials with lower thermal stability.^19^

The thermal transition behavior of the synthesized polyureas was analyzed using the DSC equipment (Figure S13). These results showed the melting temperature of PUR-1, fully aromatic polyurea, at 170 °C, which was lower than that synthesized using aliphatic diisocyanates. The melting temperature of PUR-2 was noted to be 182 °C and that of PUR-3 to be 218 °C. The DSC results of the synthesized polyurea agreed with the melting temperature range in the literature.^32^ The reason for the high melting point of PUR-3, more than that of PUR-2, can be either higher molecular weight, which could not be confirmed because of the poor solubility of polyurea, or intermolecular hydrogen bonding between chains.

Considering all of the characterization results, the feasibility of valorizing the bottom phase from PU glycolysis for the synthesis of polyureas has been proven.

#### 3.4.2 Synthesis and Characterization of Polyamides

Polyamides were synthesized by employing the recovered TDA and two chlorine-derived compounds, adipoyl chloride or isophthaloyl chloride, and by means of the two different methodologies described in Section 2.6.^20,33^

In the case of using adipoyl chloride together with TDA, an aromatic–aliphatic polyamide is obtained (Reaction 5). To the best of our knowledge, this type of material has not been previously synthesized.Polyamide synthesis employing adipoyl chloride.
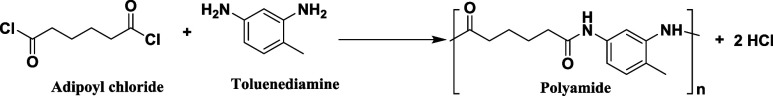
Reaction 5

When using isophthaloyl chloride and TDA, an aromatic polyamide or polyaramid is obtained, according to Reaction 6.^34^ This product is commercially known as Kevlar, has been extensively researched due to its good mechanical and thermal properties, and is also widely used in the manufacturing of optical cables, fire resistant clothing, and bullet-proof vests, among others.^35^Polyamide synthesis employing isophthaloyl chloride.
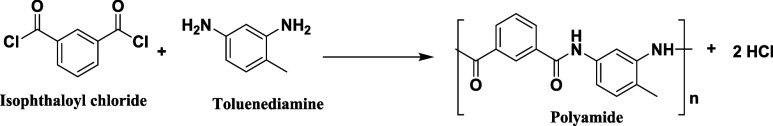
Reaction 6

In the case of the two-phase synthesis method, first, benzene was used as a solvent, as described in the literature.^33^ However, in this work, limonene (greener solvent) was tested as a substitute for benzene, allowing the synthesis of these materials by a more environmentally friendly route.

Once the synthesis was completed, the obtained solid products were purified by washing with ethanol, vacuum filtration, and drying in an oven at 50 °C for 24 h.

It should also be noted that the HCl obtained during the synthesis of these compounds was eliminated in the two-phase method by the use of NaOH in the aqueous phase, which, together with ethanol washes, ensured an adequate quality of the product obtained.

The molecular weight of the synthesized polyamides was determined by GPC (Figures S14 and S15). These results are shown in Table 3.

**Table 3:** Molecular Weights of Polyamides

adipoyl chloride		*M*_w_ (g/mol)	*M*_n_ (g/mol)	*Đ*	yield (%)
single phase		8680	6990	1.25	53
two phases	benzene	11,520	6840	1.68	56
	limonene	12,580	7360	1.62	55

isophthaloyl chloride		*M*_w_ (g/mol)	*M*_n_ (g/mol)	*Đ*	yield (%)
single phase		27,280	9970	2.74	75
two phases	benzene	14,590	3050	4.78	81
	limonene	13,930	4050	3.44	77

These results confirm the feasibility of synthesizing polyamides using the recovered toluenediamine, since the compounds presented molecular weights in an appropriate range for these commercial materials, as well as adequate conversion yields for the different compounds obtained with both methods.^36−38^ Therefore, it has been possible to demonstrate the feasibility of replacing benzene with limonene in the synthesis of these materials. The use of limonene to replace benzene gives a slightly lower yield but a lower polydispersity of the product. In the case of aromatic–aliphatic polyamides, higher *M*_n_ and *M*_w_ were achieved using limonene as a solvent. On the other hand, the implementation of limonene in the synthesis of aromatic polyamides achieved a slightly lower *M*_w_ but a lower dispersity, which can also be advantageous for limonene implementation in two-phase synthesis. The results also showed that the single-phase method is better in the synthesis of aromatic polyamides in our case. In general, aromatic polyamides are characterized by a high polydispersity, which agrees with the literature data.^39,40^ In the case of isophathaloyl, the lower reactivity of the acid chloride moiety joined to the aromatic ring seems to produce a deceleration of the initiation reaction, resulting in a broader distribution of growing chains and higher *M*_W_ and PDI than in the case of adipoyl. In addition, it can prevent or reduce any deactivation of the acid chloride group, contributing to a higher molecular weight in the single-phase case. For the two-phase case, the presence of aromatic rings within the chain leads to rapid precipitation of the polymeric chains, which causes reaction termination in a lower molecular weight and a higher PDI compared to the single-phase case.

In addition, infrared analyses were carried out to further characterize the synthesized polyamides as well as the presence of remaining unreacted portions from the starting materials. Figure S16 presents the infrared spectra of polyamides synthesized using adipoyl chloride and TDA and confirms the absence of signals from the starting materials.

The successful formation of polyamides was indicated by the disappearance of the signal at 1794 cm^–1^ corresponding to the carbonyl group of the acid chloride component and the appearance of a new signal at 1662 cm^–1^ corresponding to the amidic carbonyl group. In addition, the formation was justified by the broad signal at 3250 cm^–1^, corresponding to the N–H stretching of the amide group of the polymer, the signal at 2956 cm^–1^, corresponding to the C–H stretching of the aliphatic part, and the signals at 3044 and 1612 cm^–1^, corresponding to the C–H and C=C of the aromatic ring, respectively. Furthermore, on comparing the FTIR results of the polyamides obtained by different methodologies, either single phase or two phases using limonene or benzene, there are no significant differences, which corroborates the feasibility of replacing benzene with a green solvent such as limonene.

On the other hand, the infrared spectra of the obtained polyamides synthesized using isophthaloyl chloride and TDA are presented in Figure S17, together with the spectra of isophthaloyl chloride and TDA. As in the previous case, the disappearance of the distinctive isophthaloyl chloride signals at 1730 cm^–1^ (carbonyl group of the acid chloride) demonstrates the successful formation of aromatic polyamides.^22^ In addition, polyamide formation is confirmed by the appearance of two new signals, one at 1664 cm^–1^ corresponding to the carbonyl group of the amide and the other at 1538 cm^–1^ corresponding to N–H bending.^22^ In addition, the broad signal at 3290 cm^–1^ corresponds to the N–H stretching of the amide group of the polymer.^22^ Similarly, the spectra corresponding to the different methods do not show significant differences and therefore corroborate the viability of using limonene instead of benzene, which is a more environmentally friendly solvent.

The ^1^H NMR of aromatic–aliphatic polyamides (Figures S18, S19, and S20, 400 MHz, DMSO-*d*_6_) δ 9.81 (s, 1H) and 9.23 (s, 1H) include the two protons of the amide linkage, 7.60 (d, 1H), 7.36 (dd, 1H), and 7.07 (d,1H) correspond to the three aromatic protons of TDA incorporated in the chain, 2.23–2.41 (m, 4H) corresponds to the two symmetrical methylene groups next to the carbonyl group of the adipoyl chloride part incorporated in the chain, 2.11 (s, 3H) corresponds to the methyl group of the TDA part, and 1.62 (t, 4H) corresponds the inner two symmetrical methylene groups of adipoyl chloride. The same chemical shifts and splitting were noted for all different methods of preparations.

The ^1^H NMR of aromatic polyamide (Figure S21, 400 MHz, DMSO-*d*_6_) δ 10.48 (s, 1H) and 10.14 (s, 1H) correspond to the two protons of the amide linkage, 8.56 (t, 1H), 8.15 (m, 2H), and 7.91 (m, 1H) correspond to the aromatic protons of the isophthaloyl chloride part in the chain, 7.65 (m, 2H) and 7.26 (d, 1H) correspond to the aromatic proton of the part derived from TDA, and 2.24 (s, 3H) corresponds to the methyl group of TDA incorporated on the polymer chain. To complete the characterization of the materials obtained, TGA and DSC analyses were carried out to assess the thermal behavior of the synthesized polyamides. The TGA and DSC results are listed in Table 4.

**Table 4:** Thermal Properties of Aromatic–Aliphatic Polyamide Obtained from (a) TGA and (b) DSC

polyamide	*T*_10%_^a^ (°C)	*T*_50%_^a^ (°C)	char amount at 700 °C^a^ (%)	*T*_m_^b^ (°C)	*T*_g_^b^ (°C)
aromatic–aliphatic (Lim)	364	421	29	258	68
aromatic–aliphatic (Ben)	366	408	23	258	68
aromatic–aliphatic (single phase)	352	405	21	248	64

The TGA curves (Figure S22) showed that the degradation mechanism of the aromatic–aliphatic polyamides is the same for different syntheses. In addition, the weight loss until around 200 °C can be explained by the removal of absorbed water and solvents. The onset of thermal degradation is around 342 °C. The maximum degradation rate is around 400 °C. The difference in the char amount can be explained by the difference in molecular weight. So, in our case, the single-phase method with a lower molecular weight has a lower char amount in comparison to that of the two-phase method using limonene, which is characterized by a high molecular weight.

The DSC of aromatic–aliphatic polyamides showed similar *T*_g_ with very small differences, being independent of the solvent, and just a 4 °C difference for single-phase synthesis (Figure S23). This difference can be explained by the effect of solvent and residual HCl, especially in the case of single-phase synthesis that shows a weight loss in TGA before 200 °C due to the incorporated solvent, which causes plasticization that leads to a shift of the glass transition temperature to a lower temperature.^41,42^ Similarly, the difference in molecular weight can be the reason why there is a difference in *T*_m_ of the single-phase polyamide and two-phase polyamide using limonene.^43^

The results of the thermal characterization of the aromatic polyurea by TGA and DSC are presented in Table 5.

**Table 5:** Thermal Properties of Aromatic Polyamides Obtained from (a) TGA and (b) DSC

polyamide	*T*_10%_^a^ (°C)	*T*_50%_^a^ (°C)	char amount at 1000 °C^a^ (%)	*T*_g_^b^ (°C)
aromatic (Lim)	365	679	21	169
aromatic (Ben)	325	649	25	162
aromatic (single phase)	218	649	27	181

Due to the high thermal stability of polyamide, the TGA curves of the aromatic polyamides were run until 1000 °C, as suggested in the literature.^44^ Similar to the case of aromatic–aliphatic polyamides, the TGA curves (Figure S24) showed that all of the aromatic polyamides had the same degradation mechanism. The weight loss occurred from the beginning of heating until a temperature of about 200 °C is due to the presence of absorbed water or solvent.^29^ So, the weight loss for the single-phase synthesis may be because of DMAc entrapped within the structure of the polymer. The start of the degradation of the aromatic polyamides is around 334 °C. The maximum rate of the aromatic polyamides is around 441 °C. The difference in the char amount can be explained by the fact that the synthesized polyamides have different molecular weights; therefore, a higher molecular weight results in a higher char amount. So, the single phase has a higher char amount, and the two-phase method using limonene has a lower char amount. The DSC curves (Figure S25) of aromatic polyamides showed similar *T*_g_ for all ways of synthesis. There is only a slight difference in *T*_g_ in the range of less than 20 °C. The difference between single- and two-phase results can be explained in terms of the effect of solvent and residual HCl used in the synthesis.^41,42^ The *T*_g_ of the aromatic polyamides is in the same range as some aromatic polyamides reported in the literature.^44,45^

The TGA results have shown that both materials are thermally stable up to high temperatures, with values similar to those reported in previous studies for this type of polymer.^46,47^ The aliphatic–aromatic polyamides showed the highest decomposition rate at a temperature of 400 °C, while in the case of the aromatic polyamides, decomposition started at around 441 °C, but the highest decomposition rate occurred at higher temperatures (above 441 °C). The increased thermal stability of aromatic polyamides is in line with the properties of these materials, which justifies their use in the manufacturing of optical cables, flame-retardant clothing, and bullet-proof vests.^35^ On the other hand, the degradations corresponding to the starting materials are not observed, indicating their total transformation. Another important result to note is that char amount values indicate the feasibility of using these materials in applications with good fire-retardant properties.^48^

## 4 Conclusions

The valorization of the bottom phase of the glycolysis product has been successfully achieved, obtaining high-value-added products and demonstrating their applicability in interesting processes.

For this valorization, the conditions of bottom-phase hydrolysis have been determined: a reaction time of 3 h, a mass ratio of water to the bottom phase of 1:1, and a temperature of 200 °C, which allowed complete conversion of the carbamates into primary amines.

A vacuum distillation protocol has also been developed, allowing us to separate the recovered diethylene glycol and toluenediamine. Both recovered compounds were characterized, showing high purity comparable to that of commercial products.

The feasibility of using the recovered DEG and TDA as a glycolysis agent and a reagent, respectively, for the synthesis of other new polymers, polyureas, and polyamides, has been demonstrated.

Additionally, in the case of polyamides, the synthesis process has been improved by replacing benzene with limonene as a solvent, which is a green solvent, resulting in a more environmentally friendly synthesis process with similar products and process yields. Moreover, when adipoyl chloride was used as a reagent with the recovered TDA, an aromatic–aliphatic polyamide was obtained, which, to the best of our knowledge, has not been previously described in the literature.

## Supporting Information Available

Description of the materials and characterization techniques; infrared analyses of the glycolysis bottom phase before and after the hydrolysis process; GPC characterization of the products after the distillation process and of pure DEG and TDA; infrared characterization of different products after the distillation process; ^1^H NMR of the separated TDA; GPC chromatograms of the glycolysis product at the end time of the reaction; FTIR spectra of the formed polyurea; FTIR spectra of the starting materials for polyurea synthesis; ^1^H NMR spectrum of PUR-1, PUR-2, and PUR-3; TGA and DSC curves of the synthesized polyurea; GPC of aromatic–aliphatic polyamides; GPC of aromatic polyamides; infrared analyses of polyamides synthesized with adipoyl chloride; infrared analyses of polyamides synthesized with isophthaloyl chloride; ^1^H NMR of aliphatic–aromatic polyamides; ^1^H NMR of aromatic polyamide; and TGA and DSC curves of the synthesized aromatic–aliphatic polyamides and aromatic polyamides (PDF)

## References

[ref1] Akindoyo J. O.; Beg M. D. H.; Ghazali S.; Islam M. R.; Jeyaratnam N.; Yuvaraj A. R. Polyurethane Types, Synthesis and Applications—a Review. RSC Adv. 2016, 6 (115), 114453–114482. 10.1039/c6ra14525f.

[ref2] Simón D.; Borreguero A. M.; de Lucas A.; Gutiérrez C.; Rodríguez J. F. Sustainable Polyurethanes: Chemical Recycling to Get It. In Environment, Energy and Climate Change I; Springer, 2015; Vol. 32, pp 229–260.

[ref3] Das A.; Mahanwar P. A Brief Discussion on Advances in Polyurethane Applications. Adv. Ind. Eng. Polym. Res. 2020, 3, 93 10.1016/j.aiepr.2020.07.002.

[ref4] Kiss G.; Rusu G.; Peter F.; Tănase I.; Bandur G. Recovery of Flexible Polyurethane Foam Waste for Efficient Reuse in Industrial Formulations. Polymers 2020, 12, 1533 10.3390/polym12071533. 32664336 PMC7407941

[ref5] Nikje M. M. A.; Garmarudi A. B.; Idris A. B. Polyurethane Waste Reduction and Recycling: From Bench to Pilot Scales. Des. Monomers Polym. 2011, 14 (5), 395–421. 10.1163/138577211X587618.

[ref6] Bergmeister H.; Seyidova N.; Schreiber C.; Strobl M.; Grasl C.; Walter I.; Messner B.; Baudis S.; Fröhlich S.; Marchetti-Deschmann M.; Griesser M.; di Franco M.; Krssak M.; Liska R.; Schima H. Biodegradable, Thermoplastic Polyurethane Grafts for Small Diameter Vascular Replacements. Acta Biomater. 2015, 11, 104–113. 10.1016/j.actbio.2014.09.003. 25218664

[ref7] Mendiburu-Valor E.; Calvo-Correas T.; Martin L.; Harismendy I.; Peña-Rodriguez C.; Eceiza A. Synthesis and Characterization of Sustainable Polyurethanes from Renewable and Recycled Feedstocks. J. Cleaner Prod. 2023, 400, 136749 10.1016/j.jclepro.2023.136749.

[ref8] Fernández L. Market volume of polyurethane worldwide from 2015 to 2025, with a forecast for 2022 to 2029.

[ref9] Yuan Z.; Nag R.; Cummins E. Ranking of Potential Hazards from Microplastics Polymers in the Marine Environment. J. Hazard. Mater. 2022, 429, 128399 10.1016/j.jhazmat.2022.128399. 35236026

[ref10] Nikje M. M. A.; Tehrani Z. M. Novel Modified Nanosilica-Based on Synthesized Dipodal Silane and Its Effects on the Physical Properties of Rigid Polyurethane Foams. Des. Monomers Polym. 2010, 13, 249 10.1163/138577210X12634696333631.

[ref11] Kemona A.; Piotrowska M. Polyurethane Recycling and Disposal: Methods and Prospects. Polymers 2020, 12, 1752 10.3390/polym12081752. 32764494 PMC7464512

[ref12] Simón D.; Borreguero A. M.; de Lucas A.; Rodríguez J. F. Recycling of Polyurethanes from Laboratory to Industry, a Journey towards the Sustainability. Waste Manage. 2018, 76, 147–171. 10.1016/j.wasman.2018.03.041. 29625876

[ref13] Gausas L.; Kristensen S.; Sun H.; Ahrens A.; Donslund B.; Lindhardt A.; Skrydstrup T. Catalytic Hydrogenation of Polyurethanes to Base Chemicals: From Model Systems to Commercial and End-of-Life Polyurethane Materials. JACS Au 2021, 1, 517–524. 10.1021/jacsau.1c00050. 34467313 PMC8395660

[ref14] Yang W.; Dong Q.; Liu S.; Xie H.; Liu L.; Li J. Recycling and Disposal Methods for Polyurethane Foam Wastes. Procedia Environ. Sci. 2012, 16, 167 10.1016/j.proenv.2012.10.023.

[ref15] Gama N. V.; Ferreira A.; Barros-Timmons A. Polyurethane Foams: Past, Present, and Future. Materials 2018, 11, 1841 10.3390/ma11101841. 30262722 PMC6213201

[ref16] Heiran R.; Ghaderian A.; Reghunadhan A.; Sedaghati F.; Thomas S.; Haghighi A. hossein. Glycolysis: An Efficient Route for Recycling of End of Life Polyurethane Foams. J. Polym. Res. 2021, 28 (1), 22 10.1007/s10965-020-02383-z.

[ref17] del Amo J.; Iswar S.; Vanbergen T.; Borreguero A.; Vos D.; Verlent I.; Willems J.; Rodríguez J. Polyurethane Composites Recycling with Styrene–Acrylonitrile and Calcium Carbonate Recovery. Materials 2024, 17, 2844 10.3390/ma17122844. 38930213 PMC11204646

[ref18] Simón D.; Borreguero A. M.; de Lucas A.; Molero C.; Rodríguez J. F. Novel Polyol Initiator from Polyurethane Recycling Residue. J. Mater. Cycles Waste Manage. 2013, 16, 525–532. 10.1007/s10163-013-0205-y.

[ref19] Li X.; Chen D. Synthesis and Characterization of Aromatic/Aliphatic Co-Polyureas. J. Appl. Polym. Sci. 2008, 109, 897 10.1002/app.24913.

[ref20] Shiju J.; Al-Sagheer F.; Ahmad Z. Thermal Mechanical Properties of Graphene Nano-Composites with Kevlar-Nomex Copolymer: A Comparison of the Physical and Chemical Interactions. Polymers 2020, 12, 2740 10.3390/polym12112740. 33227943 PMC7699200

[ref21] Wittbecker E. L.; Morgan P. W. Interfacial Polycondensation. I. J. Polym. Sci. 1959, 40 (137), 289–297.

[ref22] Libretexst. Infrared Spectroscopy Absorption Table. Sonoma State University Saves Students Hard Cash with the Libretexts 2013.

[ref23] Bhabhe M. D.; Athawale V. D. Gel Permeation Chromatographic Method for Monitoring the Transesterification Reaction in a Two-Step Chemoenzymatic Synthesis of Urethane Oil Based on Vegetable Oils. J. Chromatogr. A 1995, 718, 299 10.1016/0021-9673(95)00441-6.

[ref24] Kumar N.; Gupta P. K.; Khilari S.; Ranganath K. V. S. Synthesis, Characterization and Catalytic Application of Functionalized Polyureas. J. Polym. Res. 2023, 30 (3), 1–8. 10.1007/s10965-023-03492-1.

[ref25] Shang J.; Liu S.; Ma X.; Lu L.; Deng Y. A New Route of CO2 Catalytic Activation: Syntheses of N-Substituted Carbamates from Dialkyl Carbonates and Polyureas. Green Chem. 2012, 14 (10), 2899–2906. 10.1039/c2gc36043h.

[ref26] Mattia J.; Painter P. A Comparison of Hydrogen Bonding and Order in a Polyurethane and Poly(Urethane-Urea) and Their Blends with Poly(Ethylene Glycol). Macromolecules 2007, 40 (5), 1546–1554. 10.1021/ma0626362.

[ref27] Hussien M. A.; Ashour G. R.; Albukhari S. M.; Saleh T. S.; Hussein M. A. Favorable Heteroaromatic Thiazole-Based Polyurea Derivatives as Interesting Biologically Active Products. Polymers Basel 2023, 15 (12), 2662 10.3390/polym15122662. 37376308 PMC10303549

[ref28] Wang P.; Ma X.; Li Q.; Yang B.; Shang J.; Deng Y. Green Synthesis of Polyureas from CO 2 and Diamines with a Functional Ionic Liquid as the Catalyst. RSC Adv. 2016, 6 (59), 54013–54019. 10.1039/C6RA07452A.

[ref29] Zulfiqar S.; Sarwar M. I. Synthesis and Characterization of Aromatic-Aliphatic Polyamide Nanocomposite Films Incorporating a Thermally Stable Organoclay. Nanoscale Res. Lett. 2009, 4 (5), 391–399. 10.1007/s11671-009-9258-1. 20596518 PMC2893790

[ref30] White B. T.; Migliore J. M.; Mapesa E. U.; Wolfgang J. D.; Sangoro J.; Long T. E. Isocyanate- and Solvent-Free Synthesis of Melt Processible Polyurea Elastomers Derived from Urea as a Monomer. RSC Adv. 2020, 10 (32), 18760–18768. 10.1039/D0RA02369H. 35518320 PMC9054001

[ref31] Maia F.; Tedim J.; Bastos A. C.; Ferreira M. G. S.; Zheludkevich M. L. Active Sensing Coating for Early Detection of Corrosion Processes. RSC Adv. 2014, 4 (34), 17780–17786. 10.1039/C4RA00826J.

[ref32] Shahi V.; Alizadeh V.; Amirkhizi A. V. Thermo-Mechanical Characterization of Polyurea Variants. Mech. Time-Depend. Mater. 2021, 25 (3), 447–471. 10.1007/s11043-020-09454-0.

[ref33] Beaman R. G.; Morgan P. W.; Koller C. R.; Wittbecker E. L.; Magat E. E. Interfacial Polycondensation. III. Polyamides. J. Polym. Sci. 1959, 40 (137), 329–336. 10.1002/pol.1959.1204013703.

[ref34] Greene J. P. 12—Polymer Composites. In Automotive Plastics and Composites; Greene J. P., Ed.; William Andrew Publishing, 2021; pp 191–222.

[ref35] DuPont. What is kevlar? www.dupont.es/kevlar/what-is-kevlar.html (Accessed September 02, 2024).

[ref36] Tanaka H.; Wu G.; Iwanaga Y.; Sanui K.; Ogata N. Synthesis of Polyamides by Direct Polycondensation with Picryl Chloride. II. Reaction Conditions and Mechanism. Polym. J. 1982, 14 (8), 635–642. 10.1295/polymj.14.635.

[ref37] Zeng H.; Guan Z. Direct Synthesis of Polyamides via Catalytic Dehydrogenation of Diols and Diamines. J. Am. Chem. Soc. 2011, 133, 1159–1161. 10.1021/ja106958s. 21204554 PMC3033491

[ref38] Kamran M.; Davidson M. G.; de Vos S.; Tsanaktsis V.; Yeniad B. Synthesis and Characterisation of Polyamides Based on 2,5-Furandicarboxylic Acid as a Sustainable Building Block for Engineering Plastics. Polym. Chem. 2022, 13 (23), 3433–3443. 10.1039/D2PY00189F.

[ref39] Zulfiqar S.; Ahmad Z.; Sarwar M. I. Soluble Aromatic Polyamide Bearing Ether Linkages: Synthesis and Characterization. Colloid Polym. Sci. 2007, 285 (15), 1749–1754. 10.1007/s00396-007-1768-8.

[ref40] Fraser A. C.; Hegde M.; oude Lohuis P. A. M.; Dingemans T. J. A Synthetic Strategy towards Sulfonated All-Aromatic Polyamides and Poly(Benzoxazole-Co-Amides). Polymer 2024, 290, 126569 10.1016/j.polymer.2023.126569.

[ref41] Li H.; Xiao R. Glass Transition Behavior of Wet Polymers. Materials 2021, 14 (4), 1–13. 10.3390/MA14040730. PMC7915364 33557319

[ref42] Zhang Y.; Liu B.; Wang L.-J.; Deng Y.-H.; Zhou S.-Y.; Feng J.-W. Preparation, Structure and Properties of Acid Aqueous Solution Plasticized Thermoplastic Chitosan. Polymers 2019, 11, 818 10.3390/polym11050818. 31067705 PMC6571857

[ref43] Fernández-Tena A.; Pérez-Camargo R. A.; Coulembier O.; Sangroniz L.; Aranburu N.; Guerrica-Echevarria G.; Liu G.; Wang D.; Cavallo D.; Müller A. J. Effect of Molecular Weight on the Crystallization and Melt Memory of Poly(ϵ-Caprolactone) (PCL). Macromolecules 2023, 56 (12), 4602–4620. 10.1021/acs.macromol.3c00234.

[ref44] Salunkhe P. H.; Ankushrao S. S.; Patil Y. S.; Mahindrakar J. N.; Kadam V. N.; Ubale V. P.; Ghanwat A. A. Processable Heat Resistant Polyamides Containing Tetraphenyl Thiophene Having Pendant Phenyl Moiety with Heterocyclic Quinoxaline Unit: Synthesis and Characterization. J. Macromol. Sci., Part A 2018, 55 (4), 377–383. 10.1080/10601325.2018.1444418.

[ref45] Sadavarte N. V.; Avadhani C. V.; Wadgaonkar P. P. Synthesis and Characterization of New Organosoluble Aromatic Polyamides and Polyazomethines Containing Pendent Pentadecyl Chains. High Perform. Polym. 2011, 23 (7), 494–505. 10.1177/0954008311417316.

[ref46] Mehta V.; Kumar N.; Algahtani A.; Tirth V.; Al-Mughanam T.; Chau K. Comparative Study of Chemically Treated Sugarcane and Kevlar Fiber to Develop Brake Resistance Composites. Molecules 2023, 28, 4861 10.3390/molecules28124861. 37375416 PMC10304057

[ref47] Park S.; Lee J.; Suh D.; Ju S.-Y. Synthesis and Characteristics of Novel Polyamides Having Pendent N-Phenyl Imide Groups. J. Macromol. Sci., Part A: Pure Appl.Chem. 2007, A385 & 6, 513–525. 10.1081/MA-100103364.

[ref48] Chouhan H.; Bhalla N.; Bandaru A. K.; Gebremeskel S.; Bhatnagar N. Quasi-Static and High Strain Rate Response of Kevlar Reinforced Thermoplastics. Polym. Test. 2021, 93, 106964 10.1016/j.polymertesting.2020.106964.

