# Supplementary material for: Valorization of the Isocyanate-Derived Fraction from Polyurethane Glycolysis by Synthesizing Polyureas and Polyamides
Source: ACS Sustain Chem Eng. 2024 Nov 20;12(48):17479–87. doi: 10.1021/acssuschemeng.4c05482 (PMC11615949; doi:10.1021/acssuschemeng.4c05482)
Supplement: Supplementary file 1 — sc4c05482_si_001.pdf [file sc4c05482_si_001.pdf]

# Supporting Information

**Valorization of the isocyanate-derived fraction from polyurethane glycolysis by synthesizing polyureas and polyamides**

Jesus del Amo[a], Paula Bravo[a], Mennatallah M. Alashry[a,b], Juan Tejeda[c], Juan F. Rodríguez[a], Ana M. Borreguero[a,*]

[a]*Chemical Engineering Department, University of Castilla-La Mancha, Institute of Chemical and Environmental Technology, ITQUIMA, Avda. Camilo José Cela s/n, 13004 Ciudad Real, Spain*

[b]*Chemistry Department, Faculty of Science, Mansoura University, 35516 Mansoura, Egypt.*

[c]*Área de Química Orgánica, Facultad de Ciencias y Tecnologías Químicas, Universidad de Castilla-La Mancha, 13071 Ciudad Real, Spain.*

**Corresponding author: AnaMaria.Borreguero@uclm.es*

**FIGURES**

**Figure S1.** Infrared analyses of glycolysis bottom phase before and after the hydrolysis process. (Page 5)

**Figure S2.** GPC characterization of the products after distillation process and of pure DEG and TDA. Peak I = Polyol, Peak II = Toluene dicarbamate (TDC), Peak III = Toluene carbamate amine (TCA), Peak IV = DEG and Peak V = TDA. (Page 6)

**Figure S3.** Infrared characterization of different products after distillation process and of pure diethylene glycol and toluenediamine. (Page 7)

**Figure S4.** $^1$H NMR of the separated TDA. (Page 8)

**Figure S5.** Aromatic protons of TDA. (Page 8)

**Figure S6.** GPC chromatograms of the glycolysis product at the end time of the reaction in comparison with GPC chromatograms of raw flexible polyol and DEG. Peak I = flexible polyol; Peak II-IV = reaction by-products; Peak V = DEG (Page 9)

**Figure S7**. FT-IR spectra of the formed polyurea; PUR-1 synthesized from TDI, PUR-2 From HMDI, PUR-3 is from IPDI. (Page 9)

**Figure S8.** FT-IR spectra of the starting materials for polyurea synthesis. (Page 10)

**Figure S9.** $^1$H NMR spectrum of PUR-1. (Page 10)

**Figure S10.** $^1$H NMR spectrum of PUR-2. (Page 11)
**Figure S11.** $^1$H NMR spectrum of PUR-3. (Page 12)
**Figure S12.** TGA curves of the synthesized polyurea. (Page 12)
**Figure S13.** DSC curve of the synthesized polyurea. (Page 13)
**Figure S14.** GPC of aromatic–aliphatic polyamide using a)- single-phase synthesis, b)- two-phase synthesis using Limonene, c)- two-phase synthesis using benzene. (Page 14)
**Figure S15.** GPC of aromatic polyamide using a)- single-phase synthesis, b)- two-phase synthesis using Limonene, c)- two-phase synthesis using benzene. (Page 15)
**Figure S16.** Infrared analyses of polyamides synthesized with adipoyl chloride in comparison with the spectras of starting materials. (Page 16)
**Figure S17.** Infrared analyses of polyamides synthesized with isophthaloyl chloride in comparison with the spectras of starting materials. (Page 17)
**Figure S18.** $^1$H NMR of aliphatic-aromatic polyamide using limonene in synthesis. (Page 18)
**Figure S19.** $^1$H NMR of aliphatic-aromatic polyamide using benzene in synthesis. (Page 18)
**Figure S20.** $^1$H NMR of aliphatic-aromatic polyamide using single phase in synthesis. (Page 19)
**Figure S21.** $^1$H NMR of aromatic polyamide using lemonene in synthesis. (Page 19)
**Figure S22.** TGA curves of the synthesized aromatic-aliphatic polyamides. (Page 20)
**Figure S23.** DSC curves of the synthesized aromatic-aliphatic polyamides. (Page 20)
**Figure S24.** TGA curves of the synthesized aromatic polyamides. (Page 21)
**Figure S25.** DSC curves of the synthesized aromatic polyamides. (Page 21)

## Experimental Section

### 1.1 Materials

Conventional polyurethane foams employed in the glycolysis process were supplied by Recticel in small pieces of about 1 $cm^3$. Besides, in the glycolysis process was added diethylene glycol (DEG) as glycol (purity 99.8%, supplied by Campi y Jové S.A., Barcelona, Spain) and 1,4-diazabicyclo octane[2.2.2] (DABCO) as catalyst (purity 99%, supplied by Sigma-Aldrich, Madrid, Spain). Nitrogen from air separation by the GeniSys Nitrogenerator equipment is obtained with a purity of 99%.

Mili-Q water (resistivity of 18.2 MΩ·cm) and sodium hydroxide (pure, supplied by Sigma-Aldrich, Barcelona, Spain) were employed in the hydrolysis process. The distilled product was washed using ethanol (Supplied by Guinama, Valencia, Spain).

In the synthesis of polyureas, dimethyl acetamide (DMAc) (purity 99.9%, supplied by Sigma-Aldrich, Barcelona, Spain), hexamethylene diisocyanate (HMDI) (purity 90%, supplied by Sigma-Aldrich, Barcelona, Spain), Isophorone diisocyanate (IPDI) (purity 98%, supplied by Sigma-Aldrich, Barcelona, Spain), Tolylene-2,4-diisocyanates (TDI) (purity 80%, supplied by Sigma-Aldrich, Barcelona, Spain), ethanol (Supplied by Guinama, Valencia, Spain), lithium chloride (purity 99%, supplied by Sigma-Aldich, Barcelona, Spain) and recovered toluenediamine were employed.

In polyamides synthesis, isophthaloyl chloride (purity 99%, Sigma-Aldrich, Barcelona, Spain), adipoyl chloride (purity 98%, supplied by Sigma-Aldrich, Barcelona, Spain), recovered toluenediamine, dimethyl acetamide (purity 99.9%, supplied by Sigma-Aldrich, Barcelona, Spain), acetone (purity 99.6%, supplied by Labkem, Barcelona, Spain), mili-Q water (resistivity of 18.2 MΩ·cm), sodium hydroxide (supplied by PanReac, Barcelona, Spain), benzene (purity 99.5%, supplied by PanReac, Barcelona, Spain), limonene (supplied by Almasqué, Barcelona, Spain) and ethanol (supplied by Guinama, Valencia, Spain) were used.

Finally, in characterization techniques, tetrahydrofuran (purity 99.9%, supplied by Sigma-Aldrich, Barcelona, Spain) and dimethyl acetamide (purity 99.9%, supplied by Sigma-Aldrich, Barcelona, Spain) were used. Additionally, commercial flexible polyether polyol (supplied by Repsol, Madrid, Spain) and commercial toluenediamine (purity 98%, supplied by Sigma-Aldrich, Barcelona, Spain) were used for comparison with the recovered ones.

## 1.2 Characterization techniques

### 1.2.1 Molecular weight and product composition determination by gel permeation chromatography (GPC)

The molecular weight, molecular weights distribution (MWD) and purity of the recovered products were determined by GPC analyses. The GPC equipment was a Viscotek GPCmax VE-2001 TDA 302 Detectors chromatograph with two peristaltic pumps, automatic injection system, electric

oven, two columns Water Styragel Column HR2 (pore size 500Å, molecular weight 0 to 100 g / mol) and HR0.5 (pore size 50Å, molecular weight 500 to 20,000 g/mol) and triple detection, consisting of a LALS (Low Angle Light Scattering) detector, a RALS (Right Angle Light Scattering) detector and a viscosity detector. OmniSEC 4.5.6 is the program available in the GPC equipment for recording and analysing the results. The conditions were a temperature of 40ºC, a flow rate of 1 ml/min, a sample concentration of 10 mg/ml dissolved in THF or dimethyl acetamide and an injection volume of 100 µl. Poly(ethylene glycol) standards (from Agilent) were used for MWD calibration. The area of a peak was related to the concentration of the substance in the sample by means of the response factors, which were obtained by calibrating with samples of pure substances. In order to use this characterization technique quantitatively and to calculate the purities and compositions of the different recovered products, each one of the peaks present in the chromatogram was deconvoluted. Since each of the pure compounds has been previously characterized and its retention time is known, each peak appearing in a chromatogram can be related to its compound. The intensity of the signal or area of the peak is proportional to the amount of it in the sample, so the area of each of the peaks obtained is calculated and normalized to its percentage concentration, allowing the instrument to be used quantitatively.

### 1.2.2 Fourier Transform Infrared Spectroscopy (FTIR)

Chemical structures of the recovered products or synthesized materials were determined by infrared analyses, employing a Varian 640-IR FT-IR spectrophotometer in the range of 4000 to 400 $cm^{-1}$, 8.0 $cm^{-1}$ resolution and 16 scans, with a program called Varian Resolution Pro Software, version 5.0.

### 1.2.3 Nuclear magnetic resonance ($^1H$ NMR)

The chemical structures were confirmed using NMR spectra acquired at 25 ˚C on a Bruker Avance Neo 500 or Varian 400 MHz spectrometer. The chemical shifts (δ) are recorded in ppm and are referenced to the deuterated solvent signals for $CDCl_3$ ($^1H$, δ = 7.27 ppm) and DMSO-$d_6$ ($^1H$, δ = 2.50 ppm).

### 1.2.4 Thermogravimetric analysis (TGA)

The thermal stability of the different synthesized materials was carried out by a TGA apparatus (STA 449 F3 Jupiter, NETZSCH). Samples were heated from 70 to 700 ºC or 1000 ºC at a heating rate of 5 ºC/min under nitrogen atmosphere. The obtained data were analysed using Proteus Analysis.

### 1.2.5 Differential scanning calorimetry (DSC)

Measurements of the glass transition temperature ($T_g$) of the different synthesized polymers were performed in a differential scanning calorimetry (DSC) model DSC 214 Polyma from Netzsh company. Measurements were carried out using a heating rate of 5 ºC/min. The obtained data were analysed using Proteus Analysis software.

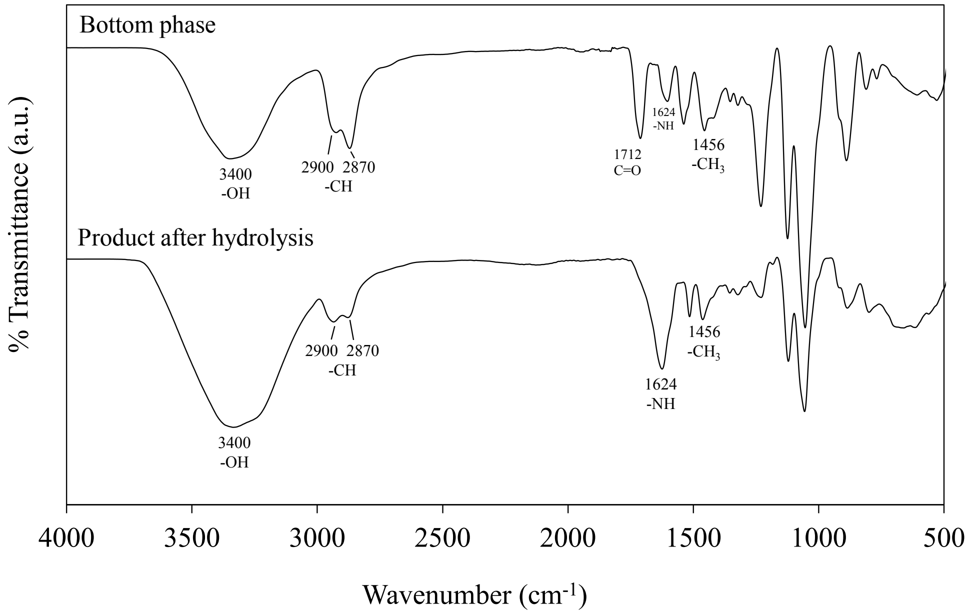


**Figure S1.** Infrared analyses of glycolysis bottom phase before and after the hydrolysis process.

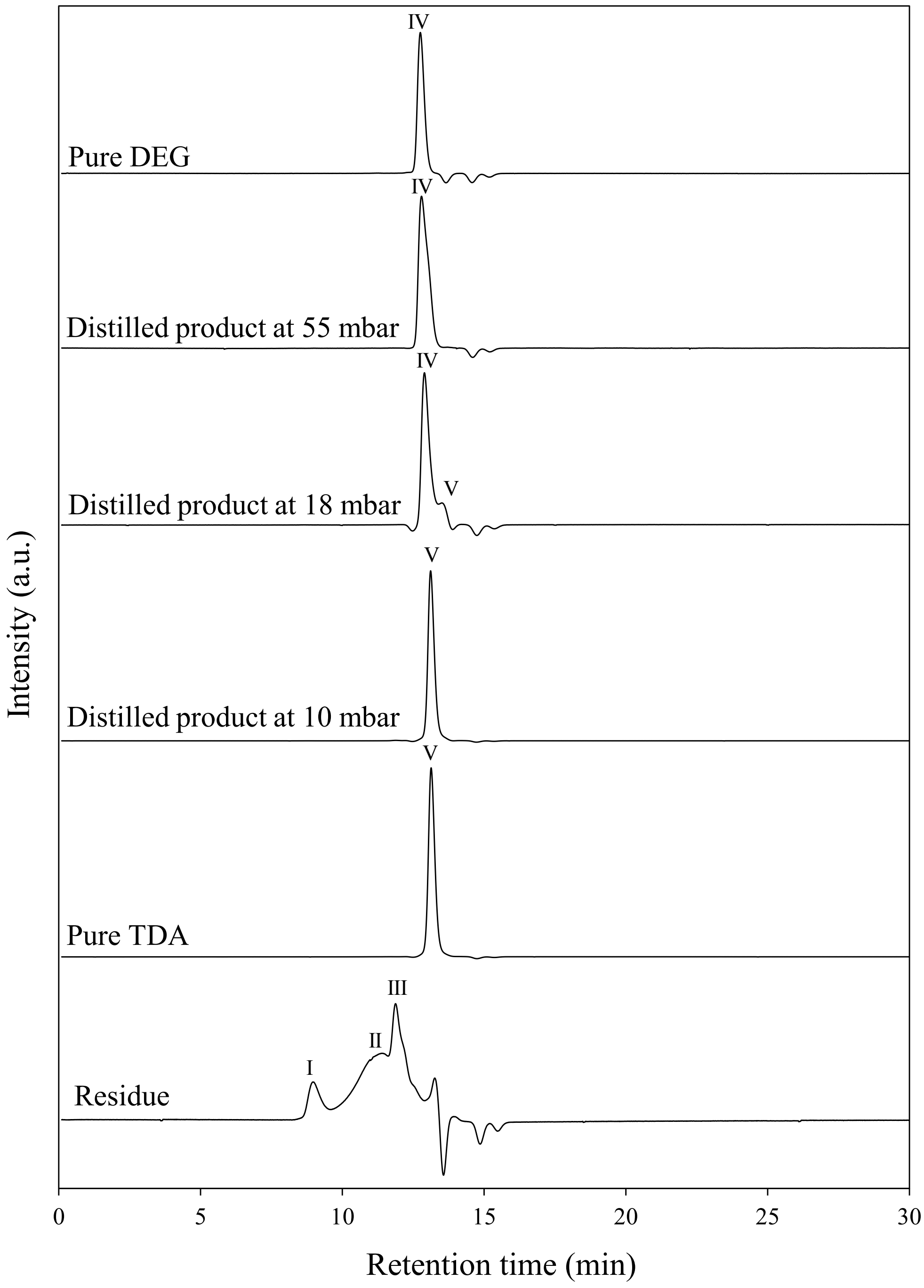


**Figure S2.** GPC characterization of the products after distillation process and of pure DEG and TDA. Peak I = Polyol, Peak II = Toluene dicarbamate (TDC), Peak III = Toluene carbamate amine (TCA), Peak IV = DEG and Peak V = TDA.

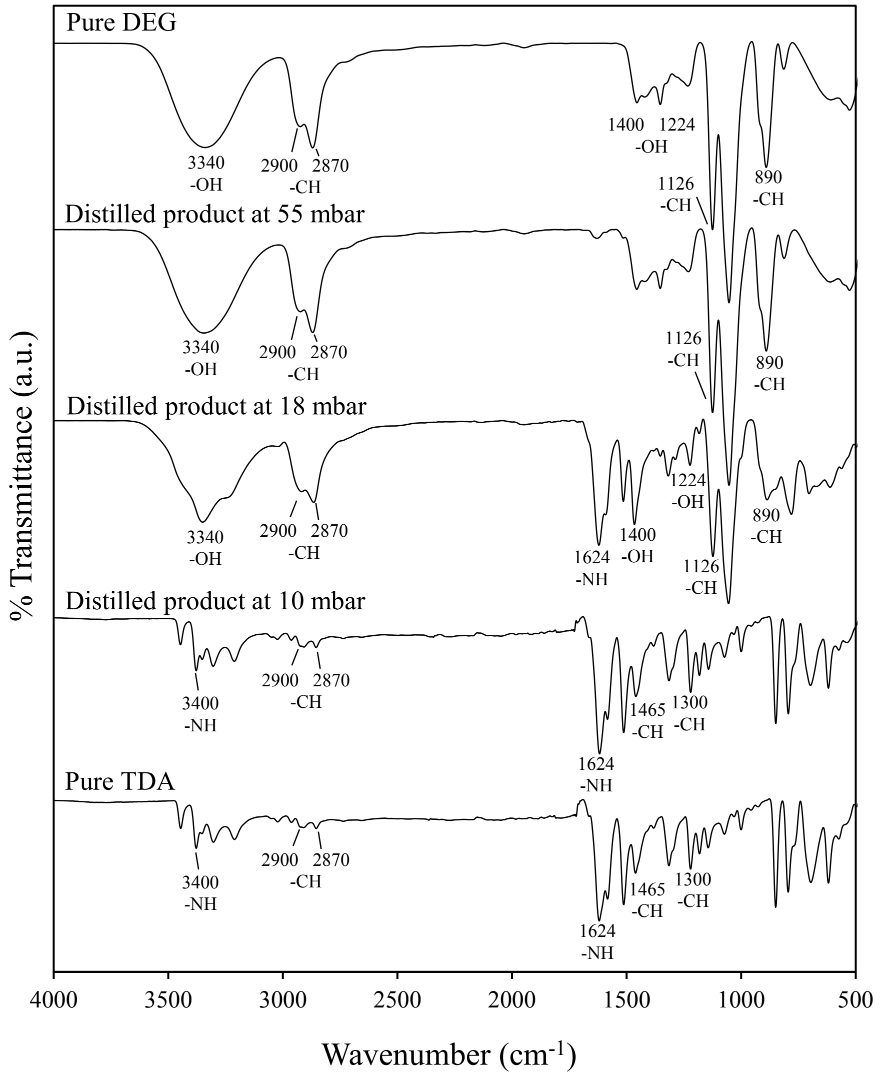


**Figure S3.** Infrared characterization of different products after distillation process and of pure diethylene glycol and toluenediamine.

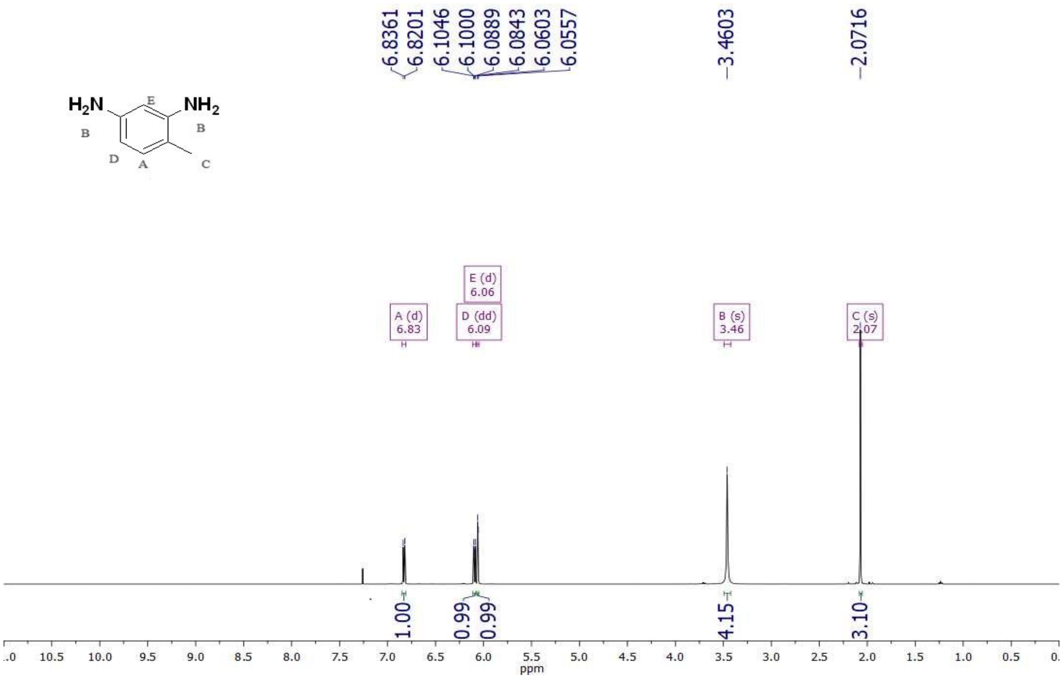


**Figure S4.** $^1$H NMR of the separated TDA.

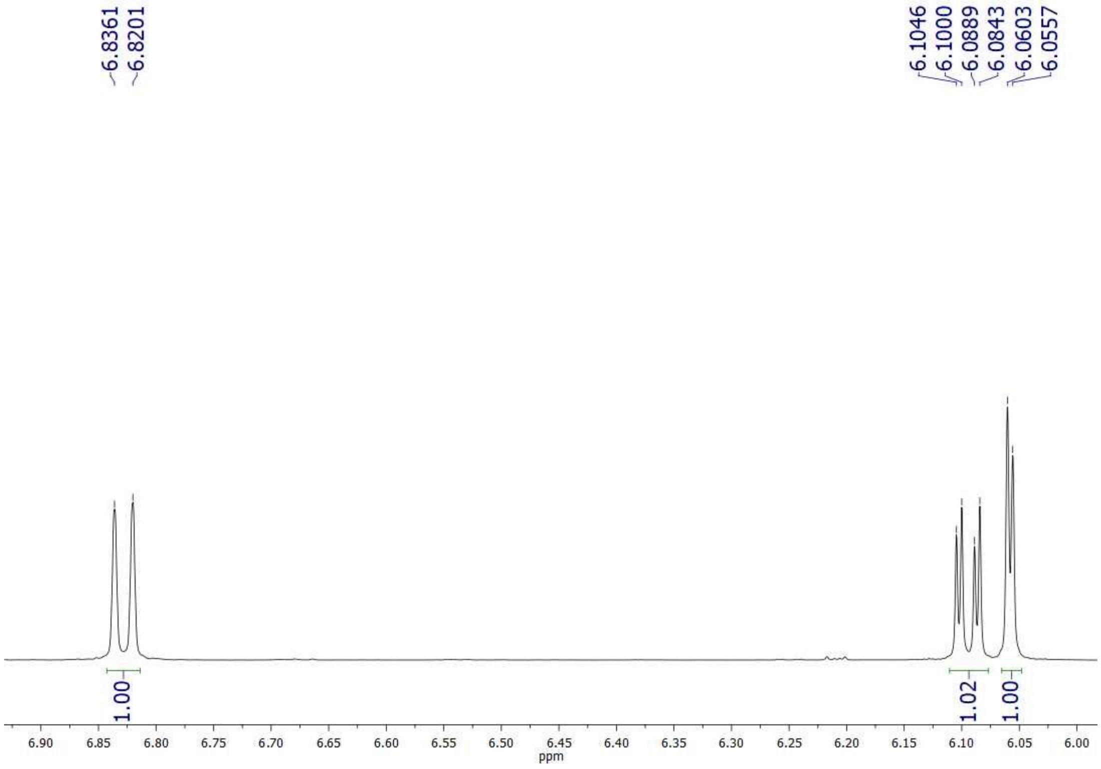


**Figure S5.** Aromatic protons of TDA.

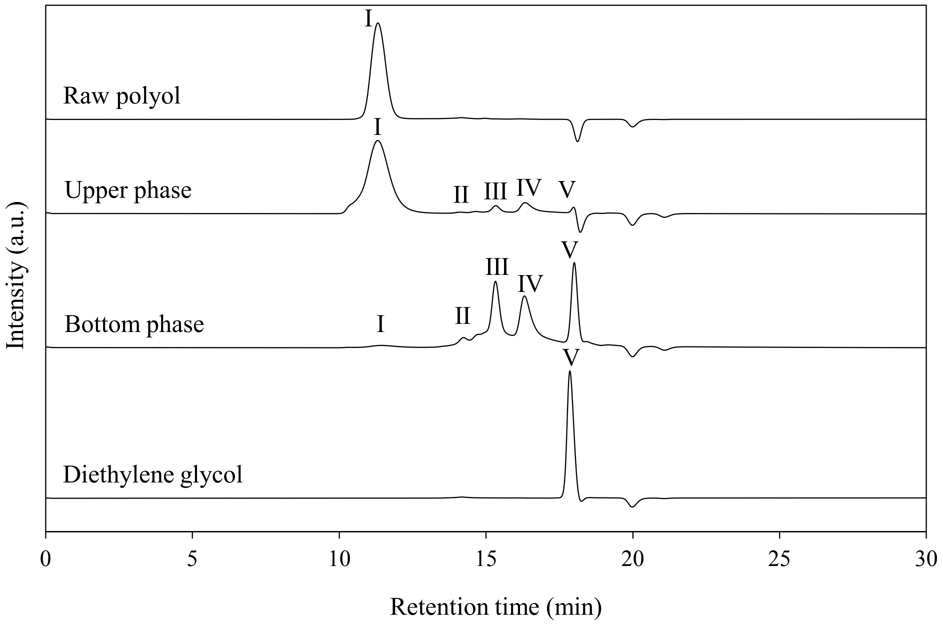


**Figure S6.** GPC chromatograms of the glycolysis product at the end time of the reaction in comparison with GPC chromatograms of raw flexible polyol and DEG. Peak I = flexible polyol; Peak II-IV = reaction by-products; Peak V = DEG

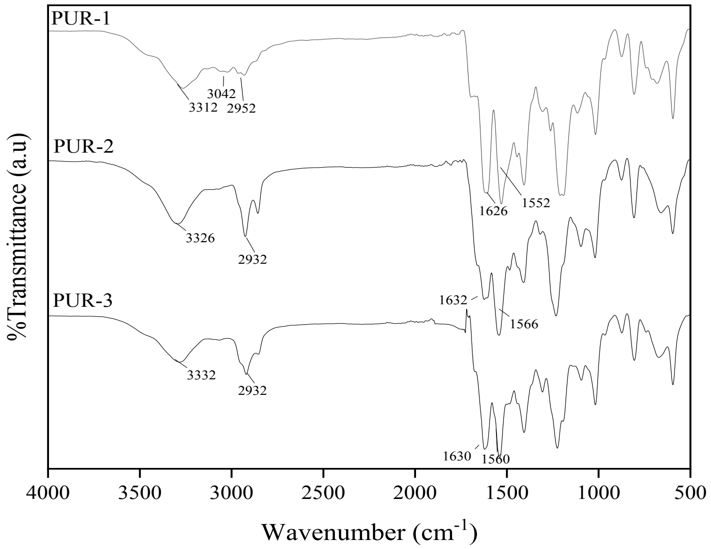


**Figure S7**. FT-IR spectra of the formed polyurea; PUR-1 synthesized from TDI, PUR-2 From HMDI, PUR-3 is from IPDI.

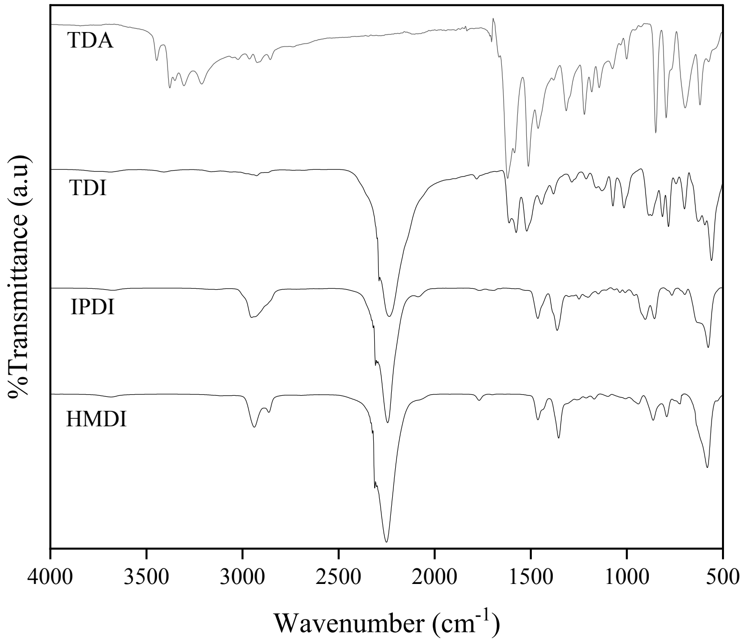


**Figure S8.** FT-IR spectra of the starting materials for polyurea synthesis.

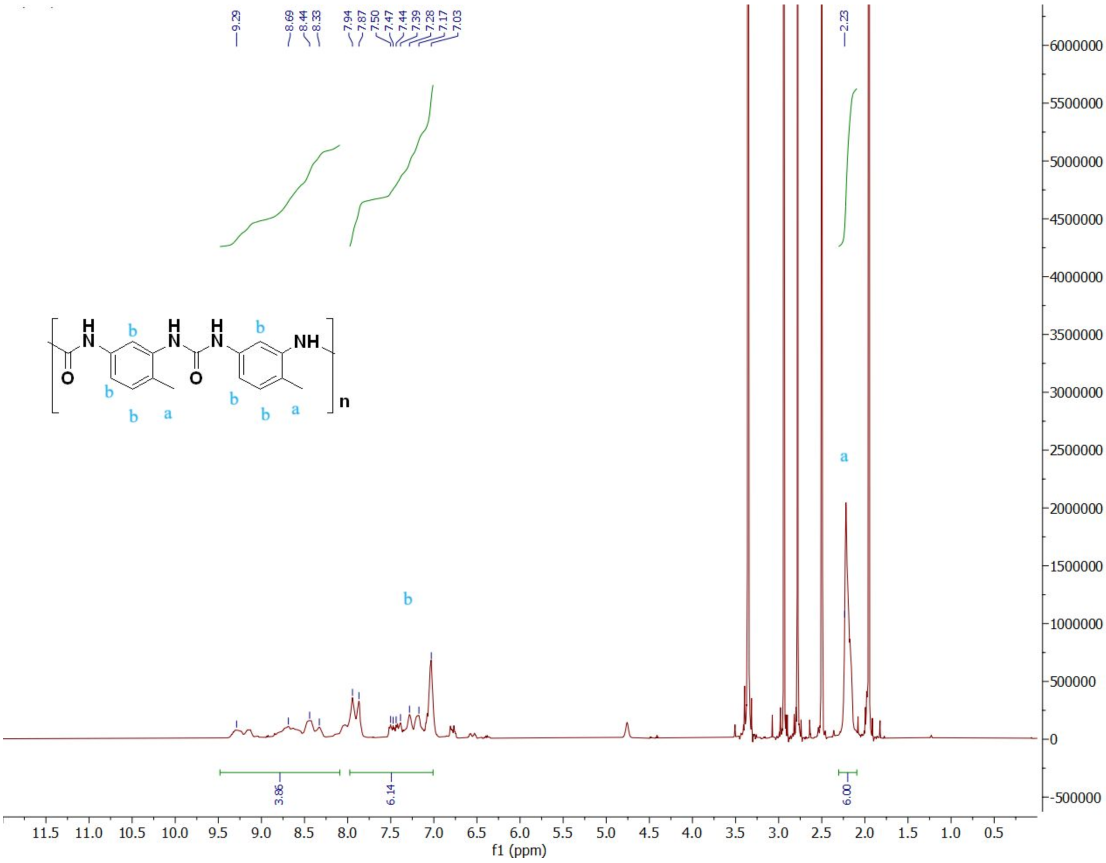


**Figure S9.** $^{1}$H NMR spectrum of PUR-1.

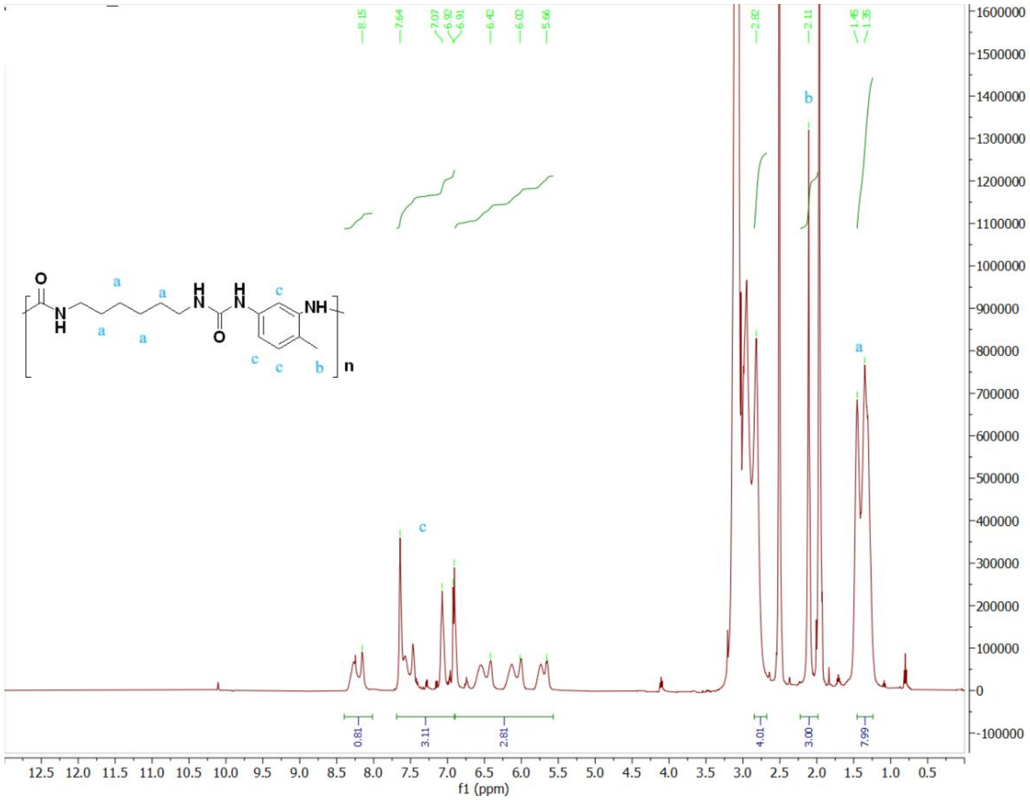


**Figure S10.** $^1$H NMR spectrum of PUR-2.

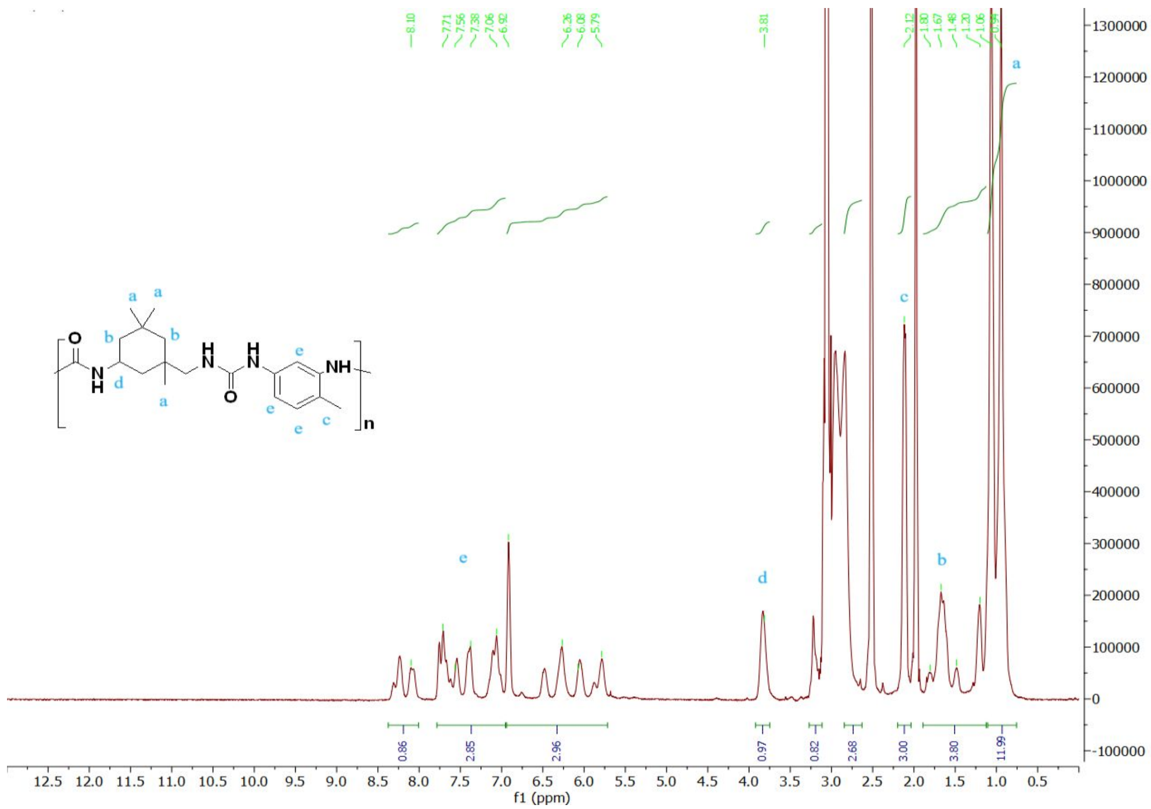


**Figure S11.** $^{1}$H NMR spectrum of PUR-3.

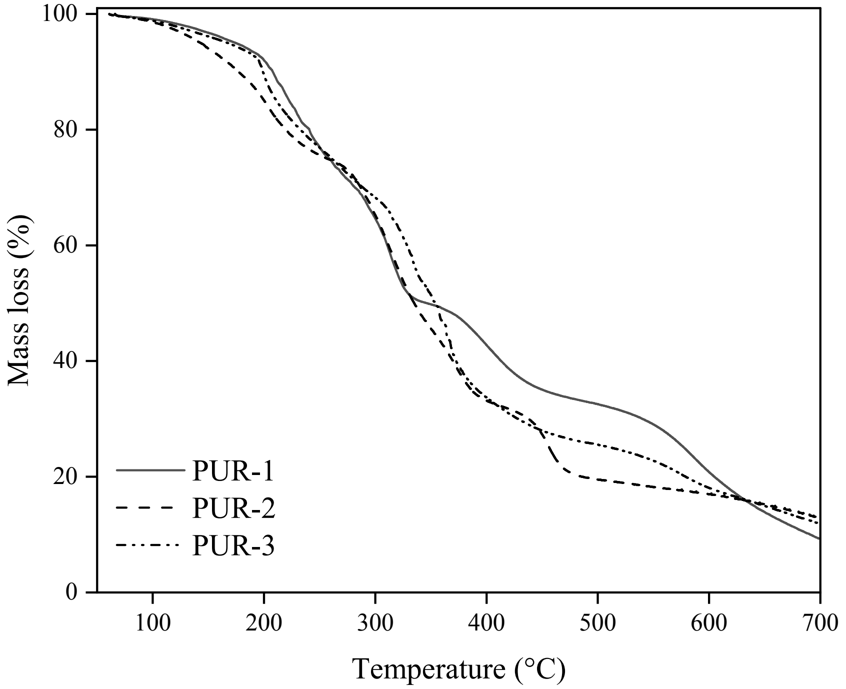


**Figure S12.** TGA curves of the synthesized polyurea.

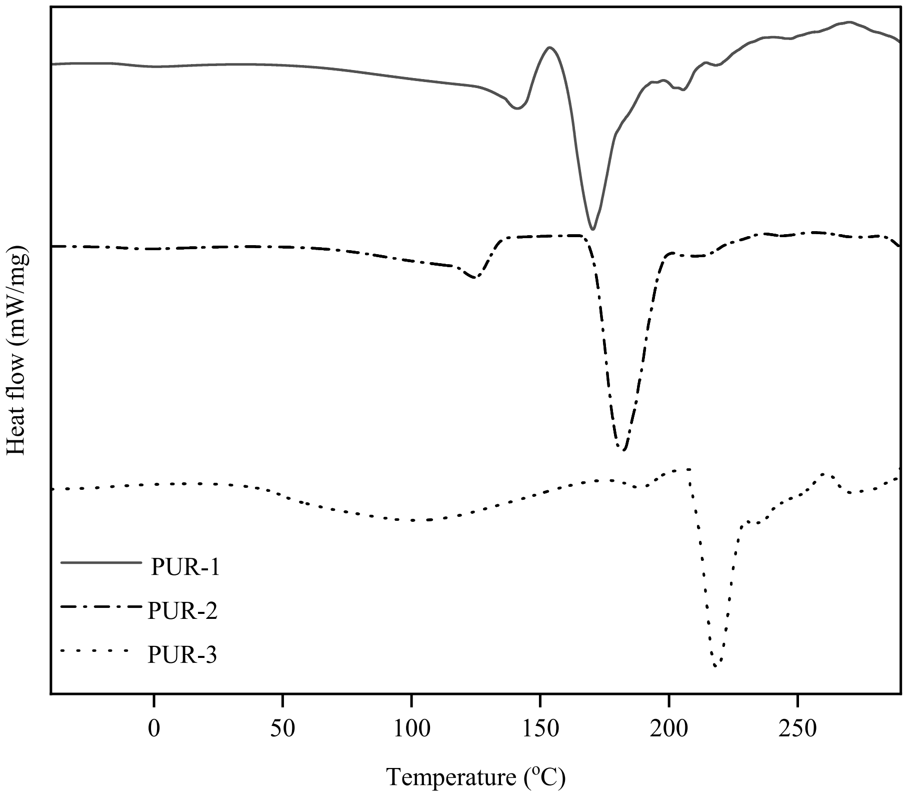


**Figure S13.** DSC curve of the synthesized polyurea.

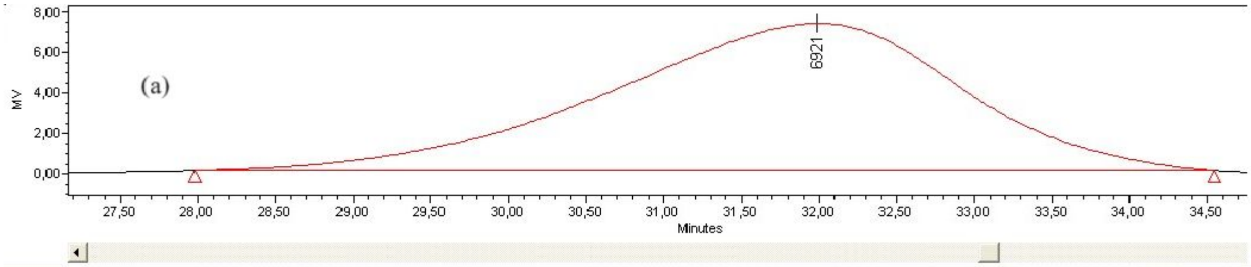


| Elution Volume (ml) | Retention Time (min) | Adjusted RT (min) | Mn (Daltons) | Mw (Daltons) | MP (Daltons) | Mv (Daltons) | Mz (Daltons) | Mz+1 (Daltons) | Polydispersity | Mz/Mw | Mz+1/Mw | K (dl/g) | alpha | Intrinsic Viscosity (dl/g) | Area (µV*sec) | % Area | Height (µV) | % Height |
|---|---|---|---|---|---|---|---|---|---|---|---|---|---|---|---|---|---|---|
| 31,990 | 31,990 | 31,990 | 6989 | 8681 | 6921 | | 10815 | 13284 | 1,242102 | 1,245837 | 1,530243 | | | | 1140971 | 100,00 | 7265 | 100,00 |

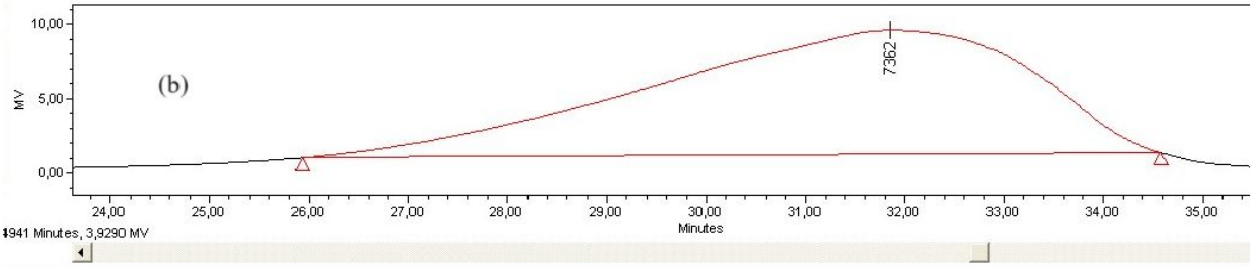


| Elution Volume (ml) | Retention Time (min) | Adjusted RT (min) | Mn (Daltons) | Mw (Daltons) | MP (Daltons) | Mv (Daltons) | Mz (Daltons) | Mz+1 (Daltons) | Polydispersity | Mz/Mw | Mz+1/Mw | K (dl/g) | alpha | Intrinsic Viscosity (dl/g) | Area (µV*sec) | % Area | Height (µV) | % Height |
|---|---|---|---|---|---|---|---|---|---|---|---|---|---|---|---|---|---|---|
| 31,853 | 31,853 | 31,853 | 7750 | 12582 | 7362 | | 20139 | 28451 | 1,623534 | 1,600576 | 2,261223 | | | | 2203056 | 100,00 | 8342 | 100,00 |

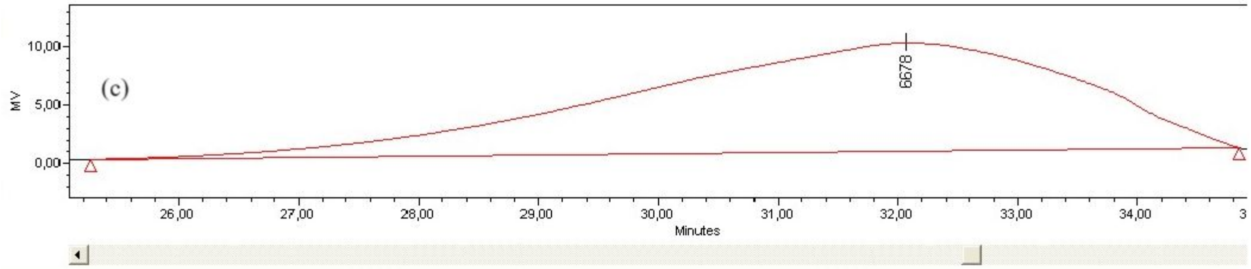


| Elution Volume (ml) | Retention Time (min) | Adjusted RT (min) | Mn (Daltons) | Mw (Daltons) | MP (Daltons) | Mv (Daltons) | Mz (Daltons) | Mz+1 (Daltons) | Polydispersity | Mz/Mw | Mz+1/Mw | K (dl/g) | alpha | Intrinsic Viscosity (dl/g) | Area (µV*sec) | % Area | Height (µV) | % Height |
|---|---|---|---|---|---|---|---|---|---|---|---|---|---|---|---|---|---|---|
| 32,069 | 32,069 | 32,069 | 6841 | 11520 | 6678 | | 19539 | 29612 | 1,683913 | 1,696100 | 2,570520 | | | | 2413512 | 100,00 | 9299 | 100,00 |

**Figure S14.** GPC of aromatic–aliphatic polyamide using a)- single-phase synthesis, b)- two-phase synthesis using Limonene, c)- two-phase synthesis using benzene.

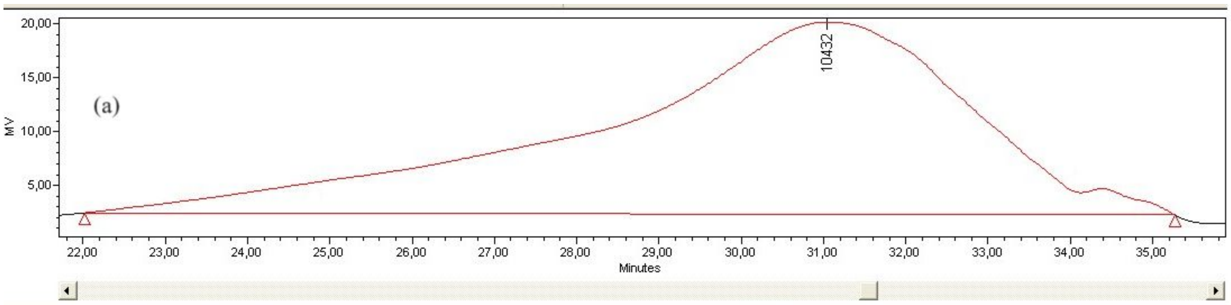


| Distribution Name | Elution Volume (ml) | Retention Time (min) | Adjusted RT (min) | Mn (Daltons) | Mw (Daltons) | MP (Daltons) | Mv (Daltons) | Mz (Daltons) | Mz+1 (Daltons) | Polydispersity | Mz/Mw | Mz+1/Mw | K (dl/g) | alpha | Intrinsic Viscosity (dl/g) | Area (µV*sec) | % Area | Height (µV) | % Height |
|---|---|---|---|---|---|---|---|---|---|---|---|---|---|---|---|---|---|---|---|
| | 31,047 | 31,047 | 31,047 | 9973 | 27284 | 10432 | | 95152 | 214753 | 2,735903 | 3,487419 | 7,870954 | | | | 5538219 | 100,00 | 17884 | 100,00 |

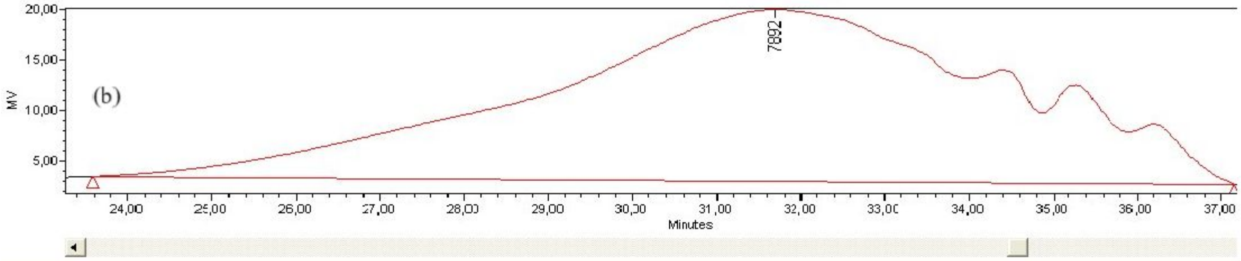


| Elution Volume (ml) | Retention Time (min) | Adjusted RT (min) | Mn (Daltons) | Mw (Daltons) | MP (Daltons) | Mv (Daltons) | Mz (Daltons) | Mz+1 (Daltons) | Polydispersity | Mz/Mw | Mz+1/Mw | K (dl/g) | alpha | Intrinsic Viscosity (dl/g) | Area (µV*sec) | % Area | Height (µV) | % Height |
|---|---|---|---|---|---|---|---|---|---|---|---|---|---|---|---|---|---|---|
| 31,696 | 31,696 | 31,696 | 4050 | 13932 | 7892 | | 36202 | 64777 | 3,439993 | 2,598559 | 4,649603 | | | | 5527783 | 100,00 | 17028 | 100,00 |

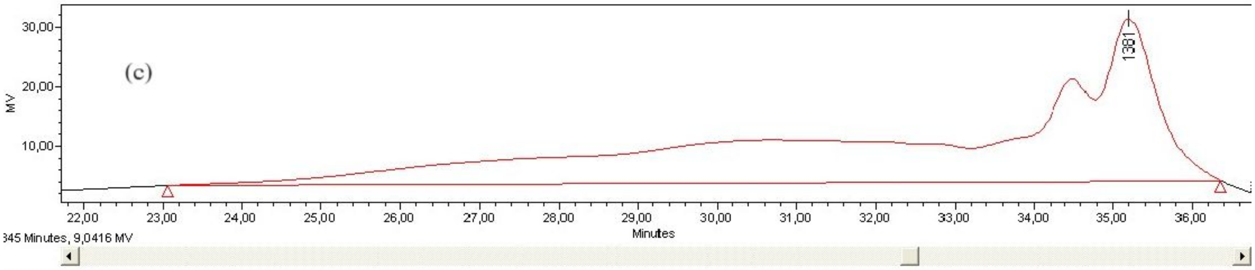


| Distribution Name | Elution Volume (ml) | Retention Time (min) | Adjusted RT (min) | Mn (Daltons) | Mw (Daltons) | MP (Daltons) | Mv (Daltons) | Mz (Daltons) | Mz+1 (Daltons) | Polydispersity | Mz/Mw | Mz+1/Mw | K (dl/g) | alpha | Intrinsic Viscosity (dl/g) | Area (µV*sec) | % Area | Height (µV) | % Height |
|---|---|---|---|---|---|---|---|---|---|---|---|---|---|---|---|---|---|---|---|
| | 35,205 | 35,205 | 35,205 | 3050 | 14591 | 1381 | | 51052 | 93119 | 4,783588 | 3,498928 | 6,382047 | | | | 4748718 | 100,00 | 27252 | 100,00 |

**Figure S15.** GPC of aromatic polyamide using a)- single-phase synthesis, b)- two-phase synthesis using Limonene, c)- two-phase synthesis using benzene.

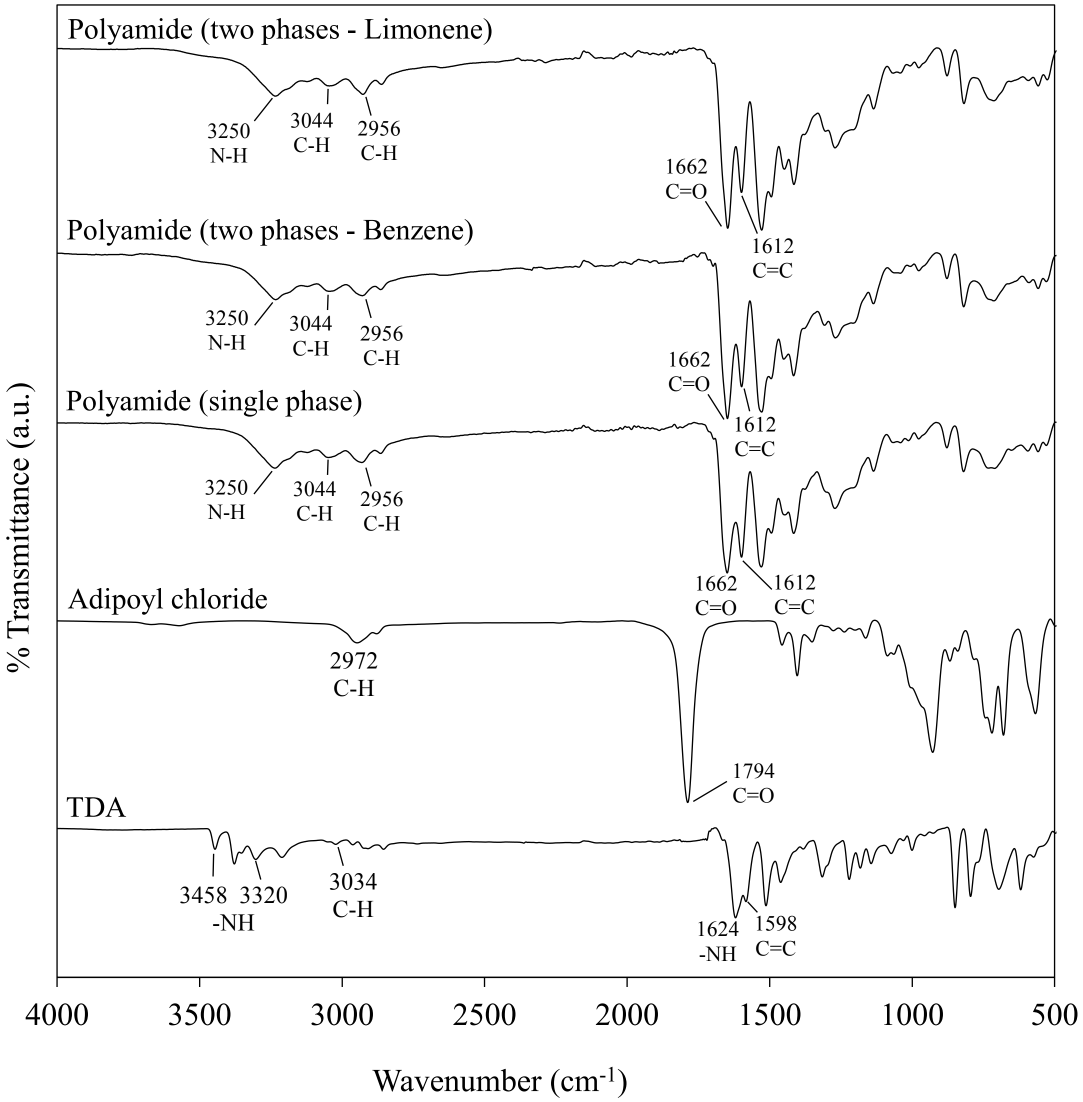


**Figure S16.** Infrared analyses of polyamides synthesized with adipoyl chloride in comparison with the spectra of starting materials.

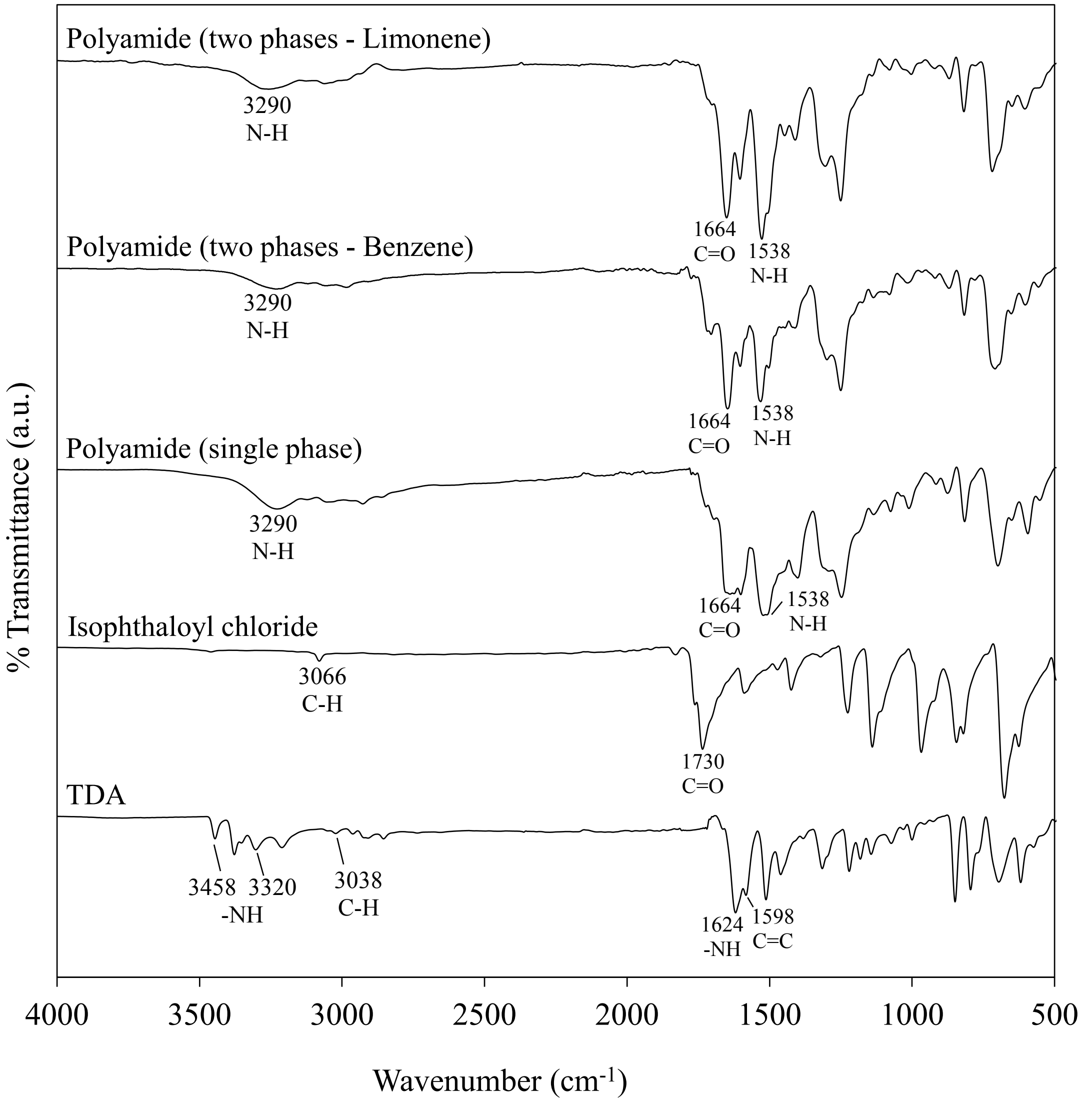


**Figure S17.** Infrared analyses of polyamides synthesized with isophthaloyl chloride in comparison with the spectra of starting materials.

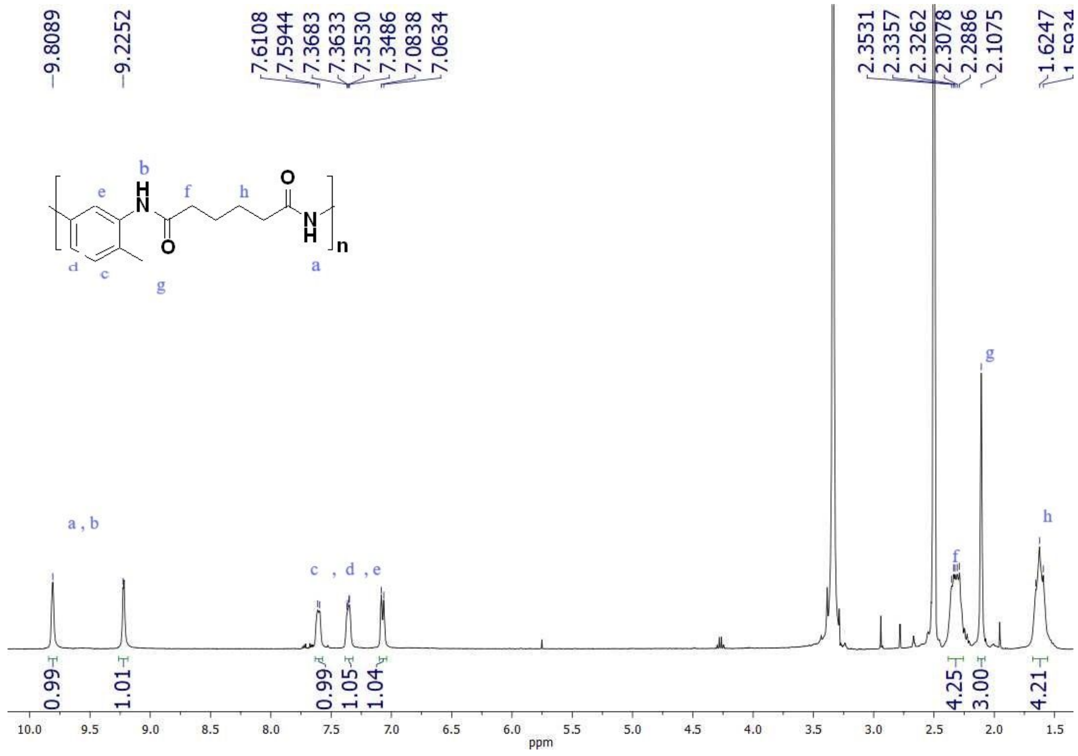


**Figure S18.** $^{1}$H NMR of aliphatic-aromatic polyamide using limonene in synthesis.

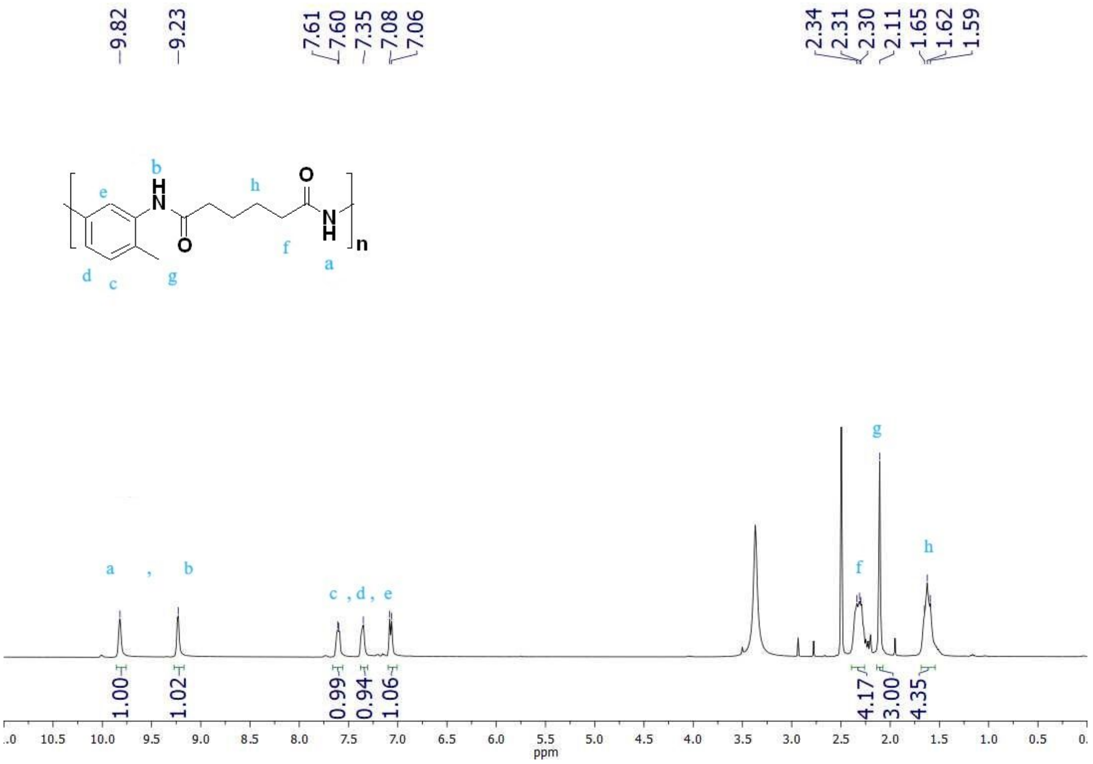


**Figure S19.** $^{1}$H NMR of aliphatic-aromatic polyamide using benzene in synthesis.

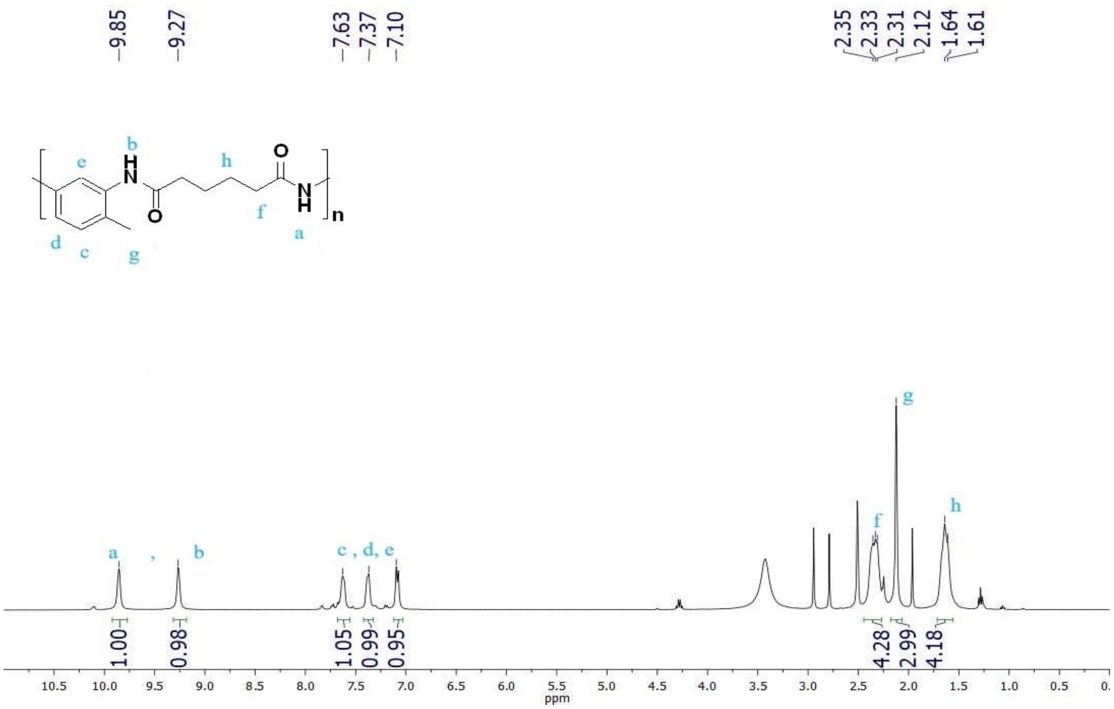


**Figure S20.** $^{1}$H NMR of aliphatic-aromatic polyamide using single phase in synthesis.

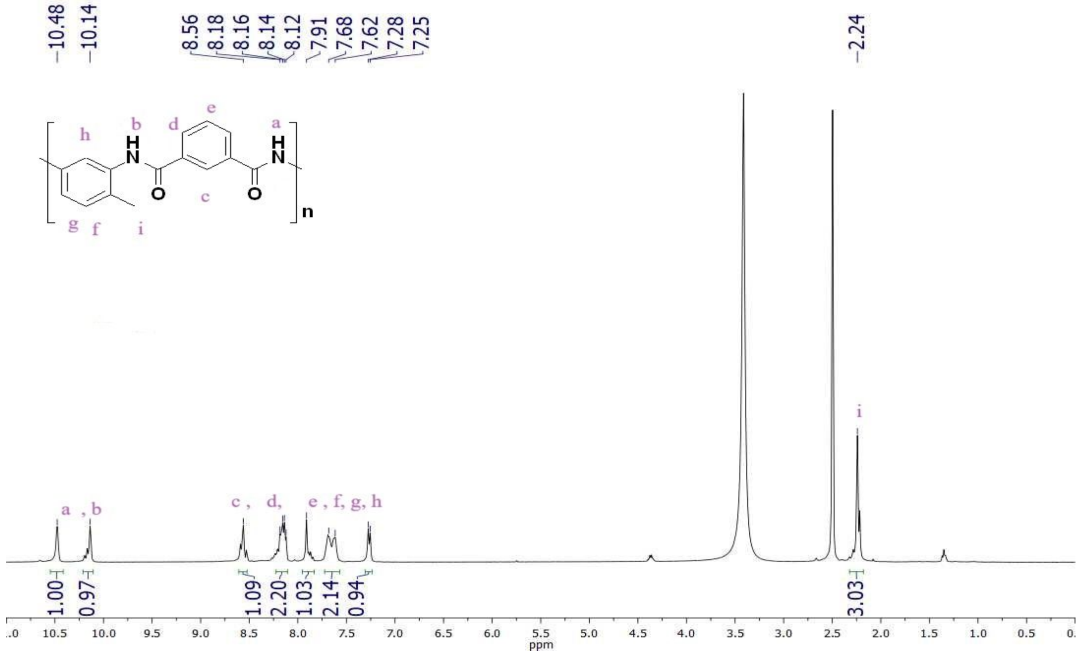


**Figure S21.** $^{1}$H NMR of aromatic polyamide using lemonene in synthesis.

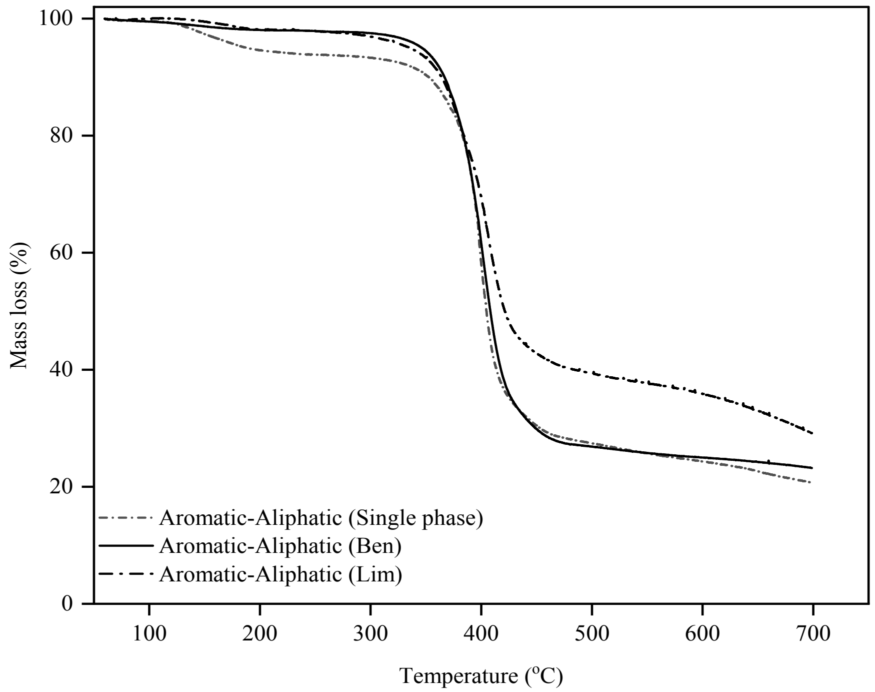


**Figure S22.** TGA curves of the synthesized aromatic-aliphatic polyamides.

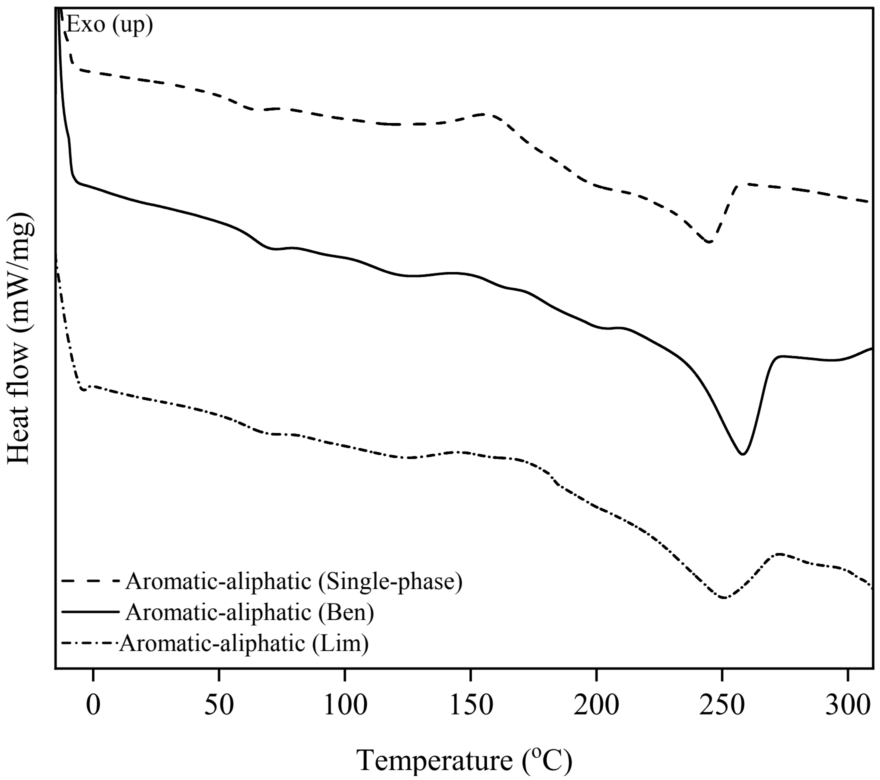


**Figure S23.** DSC curves of the synthesized aromatic-aliphatic polyamides.

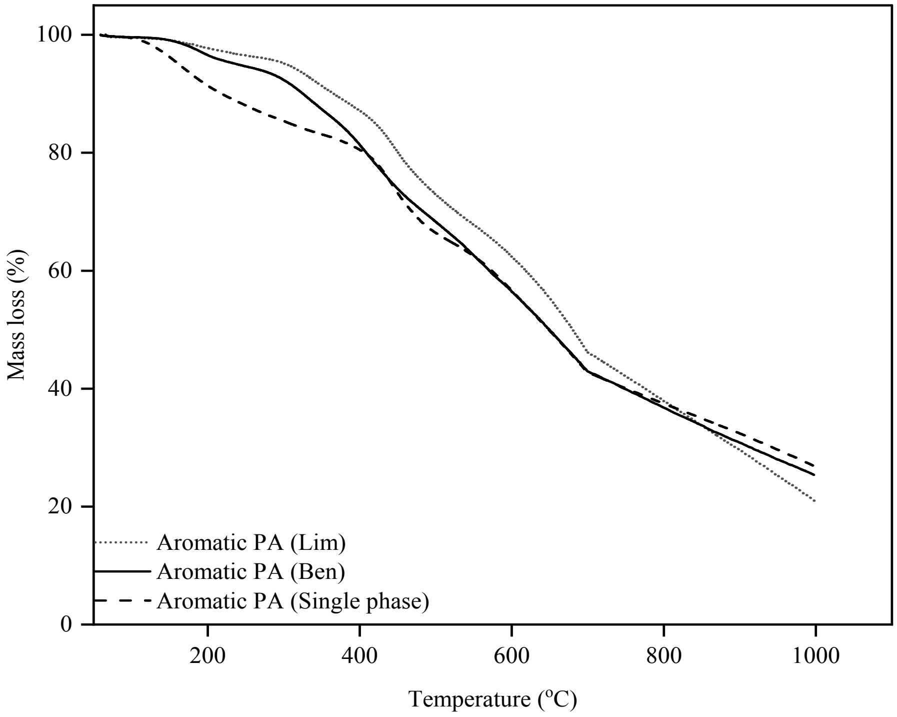


**Figure S24.** TGA curves of the synthesized aromatic polyamides.

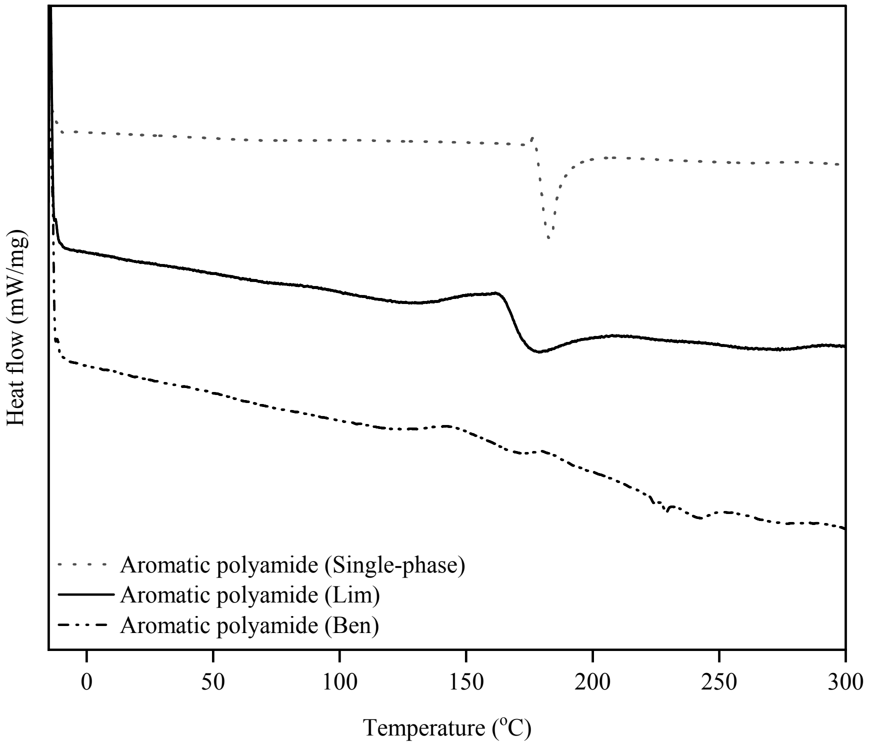


**Figure S25.** DSC curves of the synthesized aromatic polyamides.
